# ZMIZ1 enhances ERα-dependent expression of E2F2 in breast cancer

**DOI:** 10.1530/JME-23-0133

**Published:** 2024-04-25

**Authors:** Weiye Zhao, Susanna F Rose, Ryan Blake, Aňze Godicelj, Amy E Cullen, Jack Stenning, Lucy Beevors, Marcel Gehrung, Sanjeev Kumar, Kamal Kishore, Ashley Sawle, Matthew Eldridge, Federico M Giorgi, Katherine S Bridge, Florian Markowetz, Andrew N Holding

**Affiliations:** 1 Department of Biology, University of York, York, UK; 2 CRUK Cambridge Institute, University of Cambridge, Cambridge, UK; 3 Department of Cancer Immunology and Virology, Dana-Farber Cancer Institute, Smith Building, Boston, Massachusetts, USA; 4 The Institute of Metabolism and Systems Research (IMSR), University of Birmingham, College of Medical and Dental Sciences, Birmingham, UK; 5 Chris O’Brien Lifehouse, Sydney, New South Wales, Australia; 6 Department of Pharmacy and Biotechnology, University of Bologna, Bologna, Italy; 7 York Biomedical Research Institute, University of York, York, UK; 8 The Alan Turing Institute, Kings Cross, London, UK

**Keywords:** breast cancer, cancer, co-factors transcription nuclear receptors signalling patient outcome, E2F2, estrogen receptor, ZMIZ1

## Abstract

The estrogen receptor-α (ER) drives 75% of breast cancers. On activation, the ER recruits and assembles a 1–2 MDa transcriptionally active complex. These complexes can modulate tumour growth, and understanding the roles of individual proteins within these complexes can help identify new therapeutic targets. Here, we present the discovery of ER and ZMIZ1 within the same multi-protein assembly by quantitative proteomics, and validated by proximity ligation assay. We characterise ZMIZ1 function by demonstrating a significant decrease in the proliferation of ER-positive cancer cell lines. To establish a role for the ER-ZMIZ1 interaction, we measured the transcriptional changes in the estrogen response post-ZMIZ1 knockdown using an RNA-seq time-course over 24 h. Gene set enrichment analysis of the ZMIZ1-knockdown data identified a specific delay in the response of estradiol-induced cell cycle genes. Integration of ENCODE data with our RNA-seq results identified that ER and ZMIZ1 both bind the promoter of E2F2. We therefore propose that ER and ZMIZ1 interact to enable the efficient estrogenic response at subset of cell cycle genes via a novel ZMIZ1–ER–E2F2 signalling axis. Finally, we show that high ZMIZ1 expression is predictive of worse patient outcome, ER and ZMIZ1 are co-expressed in breast cancer patients in TCGA and METABRIC, and the proteins are co-localised within the nuclei of tumour cell in patient biopsies. In conclusion, we establish that ZMIZ1 is a regulator of the estrogenic cell cycle response and provide evidence of the biological importance of the ER–ZMIZ1 interaction in ER-positive patient tumours, supporting potential clinical relevance.

## Introduction

Approximately 75% of breast cancers are classified as estrogen receptor-α (ER) positive. In these cancers, the ER is no longer correctly regulated, subverts cell division regulation, and becomes the driving transcription factor in the tumour (Mohammed *et al.* 2013). Only a few primary breast cancers have mutations in the ER (Oesterreich & Davidson 2013), yet the transcriptional activity is frequently abnormal (Carroll 2016). In many metastatic tumours, the ER is still active and drives the growth of the tumour with a reduced dependence on estrogen or in a ligand-independent manner (Robinson *et al.* 2013, Toy *et al.* 2013). This critical role for the ER in disease progression has therefore made the protein a key target for therapeutics such as fulvestrant (Osborne *et al.* 2004) and tamoxifen (Jordan 2003). More recently, tamoxifen has been prescribed as a preventative treatment in high-risk healthy patients to successfully reduce their chances of developing breast cancer (Cuzick *et al.* 2015). Most women benefit from endocrine therapy with 50–70% of cases responding to treatment. However, relapse is common with the risk ranging from 10% to 41% (Pan *et al.* 2017).

### The ER transcriptional complex

One strategy to overcome relapse focuses on protein that function with or alter the transcriptional response of the ER to estrogen. The majority of ER-binding sites are at distal enhancer elements (Carroll *et al.* 2005). On binding to these sites, the receptor catalyses the formation of chromatin loops and recruits a wide range of proteins along with the mediator complex to form complexes up to 1–2 MDa in size (Liu *et al.* 2014). Through these interactions, the ER is able to facilitate the activation of RNA polymerase II at the promoters of target genes (Murakami *et al.* 2017). Without the coordination of the ER and the formation of these complexes, it is not possible for the efficient transcription of target genes to occur.

Further characterisation of ER-interacting proteins is therefore essential to identify targets for novel treatment strategies. Successful examples of cofactor-based approaches include studies demonstrating that the GREB1–ER–EZH2 transcriptional axis is directly involved in tamoxifen resistance (Wu *et al.* 2018) or identifying the pioneer-factor FOXA1 as a key opportunity for future interventions (Nakshatri & Badve 2007, Behan *et al.* 2019).

This study aims to lay the groundwork for future targeted therapies by presenting proteomic and genomic evidence that ZMIZ1 is co-expressed with ER in patient tumours and that the inhibition in ZMIZ1 reduces the early transcriptional response of cell cycle genes to estrogen in ER-positive breast cancer cells via a novel ZMIZ1–ER–E2F2 signalling axis.

## Materials and methods

### Cell lines and general cell culture

MCF7, T47D (ER-positive cell lines) and MDA-MB-231 (ER-negative cell lines) were obtained from ATCC. All cells were routinely cultured in DMEM with high glucose and pyruvate (Gibco, 41966-029) supplemented with 10% fetal bovine serum (FBS). Cells were confirmed to be mycoplasma-free using the MycoStrip test supplied by InvivoGen. Cell line authentication was confirmed by short tandem repeat genetic profiling carried out by Eurofins. All cell lines were used at less than 35 passages for all experimental work described below.

### Quantitative multiPLEXed rapid immunoprecipitation mass spectrometry of endogenous proteins

Quantitative multiPLEXed rapid immunoprecipitation mass spectrometry of endogenous proteins (qPLEX-RIME) samples were prepared as previously reported (Papachristou *et al.* 2018). Cells were grown in estrogen-free culture as previously described (Guertin *et al.* 2018). In summary, cells were cultured for 4 days in phenol red-free DMEM (Gibco) with 10% charcoal-stripped FBS and glutamine, cells were washed with PBS and media was changed daily to remove residual estrogen. Cells were cross-linked for qPLEX-RIME 45 min after the addition of 100 nM estradiol (E2) (Sigma-Aldrich) or vehicle control (ethanol).

### Proximity ligation assay

Cell lines were seeded into eight-well chamber slides in estrogen-rich complete media (DMEM supplemented with 10% FBS and glutamine), as previously described by Mohammed *et al.* (2015). After 48 h (around 50% confluence) cells were treated with 100 nM fulvestrant (Cayman 10011269) or vehicle control (ethanol). For knockdown experiments, cells were transfected using a reverse siRNA transfection with Lipofectamine RNAiMAX Transfection Reagent (Invitrogen, 13778075) and the SMARTpool on-target siZMIZ1 siRNAs along with the matched siCTRL (Dharmacon). After 24 h of treatment with fulvestrant or 48 h with siRNA, cells were rinsed in PBS and fixed with ice-cold methanol. The PLA assay (Sigma-Aldrich) was then performed according to the manufacturer’s instructions with anti-ER antibody supplied by Abcam (Ab3575) diluted at 1:400 and anti-ZMIZ1 supplied by Santa Cruz (SC-376825) diluted at 1:250. Dual antibody recognition of ER using anti-ER antibody from Abcam (Ab3575) at 1:400 dilution and anti-ER antibody from Santa Cruz (SC8002) at 1:250 was used as a positive control. An isotype anti-IgG antibody from Cell Signaling (27295) diluted to 1:250 was used, together with the anti-ER antibody from Santa Cruz antibody at 1:250, as a negative control. Following DUOLINK staining, the samples were imaged using a LSM780 confocal microscope at x630mag, with the exception of the siZMIZ1 knockdown samples and controls which were imaged with a Zeiss AXIO Observer 7 fluorescence microscope. Two replicate wells were prepared for each sample condition and four different fields of view were imaged from each sample well. Images were then analysed using Fiji software (Schindelin *et al.* 2012). The number of PLA signals (fluorescent dots) to number of cells per image was assessed using the ‘Analyse particle’ function. The statistical analysis was conducted using a Student’s *t*-test, followed by Bonferroni adjustment for multiple comparisons.

### Cell growth assay

MCF7, T47D, and MDA-MB-231 cell growth was undertaken in estrogen-rich complete media with 10% FBS and glutamine, monitored by incucyte. siRNA knockdown was undertaken with SMARTpool on-target siZMIZ1 siRNAs along with the matched siCTRL (Dharmacon) and was transfected with Lipofectamine RNAiMAX reagent (Thermo Fisher Scientific) according to the manufacturer’s protocol. Cells were reverse transfected, and media was refreshed after 24 h to minimise the toxicity of the transfection reagents.

Knockdown was confirmed by RT-qPCR and western blotting.

### Western blotting of ZMIZ1

Western blotting was performed in accordance with the General Protocol for Western Blotting provided by Bio-Rad with certain modifications. SDS-PAGE was carried out using 4–20% Mini-PROTEAN® TGX™ Precast Gels from Bio-Rad. Proteins were transferred from the gel to a PVDF membrane in the iBlot 2 Transfer Stacks (Thermo Fisher, IB24001) using the iBlot 2 Gel Transfer Device.

Five percent (w/v) fat-free milk in TBST buffer was used as the blocking buffer. Primary and secondary antibodies were appropriately diluted using the blocking buffer. Antibodies were as follows: RAI17/ZMIZ1 (E2X3X, 1:1000) rabbit mAb (Cell Signaling Technology, 89500), anti-b-Actin antibody (Sigma A1978, 1:5000), peroxidase AffiniPure goat anti-rabbit IgG (Jackson ImmnuoResearch 111-035-144, 1:7500), peroxidase AffiniPure goat anti-mouse IgG (Jackson ImmnuoResearch 115-035-174, 1:7500). Chemiluminescent imaging was performed using SuperSignal West Pico PLUS Chemiluminescent Substrate (Thermo Fisher, 34579) with an iBright imaging system (Thermo Fisher).

### RNA-sequencing and GSEA

Cell growth was undertaken in phenol red-free DMEM + 10% charcoal-stripped FBS. siRNA knockdown was undertaken with SMARTpool ontarget siZMIZ1 along with the matched siCTRL (Dharmacon) and was transfected with Lipofectamine RNAiMAX reagent (Thermo Fisher Scientific). About 24 h after transfection, the media was refreshed, and stimulated with 100 nM E2 or vehicle control. After 3, 6, 12 and 24 h stimulation with either E2 or ethanol, RNA was collected for four biological replicates per cell line and libraries were prepared by TruSeq mRNA Library Prep (Illumina). The libraries were sequenced on the Illumina HiSeq 4000 platform. Reads were aligned with HiSat2 to hg19 and count matrix established with HtseqCount v2.1.0. Differential analysis of expression was undertaken with the R-package DESeq2 (Love *et al.* 2014) and GSEA (Mootha *et al.* 2003, Subramanian *et al.* 2005) was undertaken using the implementation in VULCAN (Holding *et al.* 2019).

### Chromatin immunoprecipitation–quantitative real-time PCR

ChIP-qPCR was undertaken according to the published methods (Glont *et al.* 2019). Five micrograms anti-ZMIZ1 antibody (Abnova, PAB4820) or normal rabbit IgG (Cell Signaling Technology, 2729) was used for each ChIP reaction.

The following primers were used for probing the promoter of E2F2 (forward 5′-CAGCTTGGGAGAGTAGAAGAAC, reverse 5′-CCAAGGTCATACAGAGAGATTCC). qPCRs were set up with Fast SYBR Green Master Mix (Thermo Fisher Scientific, 4385612) per the users’ manual. Technical triplicate was prepared for each reaction. All the qPCR assays were conducted in an Admiral QuantStudio 3 Real-Time PCR System (Applied BioSystems) under the default fast, comparative CT thermo profile. Statistical analysis was performed using paired *t*-test with the alternative hypothesis that ZMIZ1 ChIP enrichment would decrease on fulvestrant treatment.

### Survival analysis

Initial survival analyses were undertaken using KMplot (Gyorffy 2021). Patients were stratified on ZMIZ1 expression with median cut-off selected. Recurrence-free survival (RFS) was used as outcome. ER-positive status was established from array data. These settings on 1 August 2023 gave *P*-value *<*0.01 for ER-positive (*n* = 5526) patients and not significant for ER-negative (*n* = 2009). For the comparison of treatment outcomes, either tamoxifen only (*n* = 2038, *P* = 0.0051) or excluding endocrine therapy (*n* = 1063, *P* = 0.27) was selected. METABRIC and TCGA survival data was downloaded from cBioPortal (Cerami *et al.* 2012), analysis was undertaken in R, the most suitable cut-off for expression was calculated using ‘survminer’ package and data was fit using the ‘survival’ package.

### Network analysis of ER and ZMIZ1 activity

Expression data from TCGA and METABRIC was analysed using VIPER (Alvarez *et al.* 2016) and ARACNe-derived networks (Lachmann *et al.* 2016) from the R-package ‘aracne.networks’ in Bioconductor. Statistical analysis and plotting of results were undertaken in R.

### Immunohistochemistry

Antibody specificity was confirmed on formalin-fixed cell pellets of MCF7 cells (Supplementary Fig. 1, see section on supplementary materials given at the end of this article). Patient tissue samples were run on Leica’s Polymer Refine Detection System (DS9800) using their standard template on the automated Bond-III platform. MCF7 cells were cultured in estrogen-rich complete DMEM media with 10% BS and glutamine. Post-siRNA knockdown of ZMIZ1 were used as negative control to ensure antibody specific. Dewaxing and re-hydration prior to IHC were automated on the Leica ST5020, along with the post-IHC dehydration and clearing. Sections were mounted using Leica’s coverslipper CV5030. The specific antibody targeting ZMIZ1 was purchased from R&D Systems (AF8107) and used at a concentration of 2 µg/mL (1:250 dilution). The sodium citrate pre-treatment was run at 100˚C. The secondary (post-primary) was rabbit anti-sheep from Jackson ImmunoResearch (r313-005-003), diluted 1:500. DAB Enhancer was included as an ancillary reagent (Leica, AR9432).

Patient tissue samples were processed as described for the MCF7 fixed cell pellets. ER-α antibody was purchased from Novacastra (NCL-ER-6F11/2) and samples processed as previously described (Bruna *et al.* 2016).

## Results

### qPLEX-RIME of ER activation identifies novel co-factor ZMIZ1

**Figure 1:**
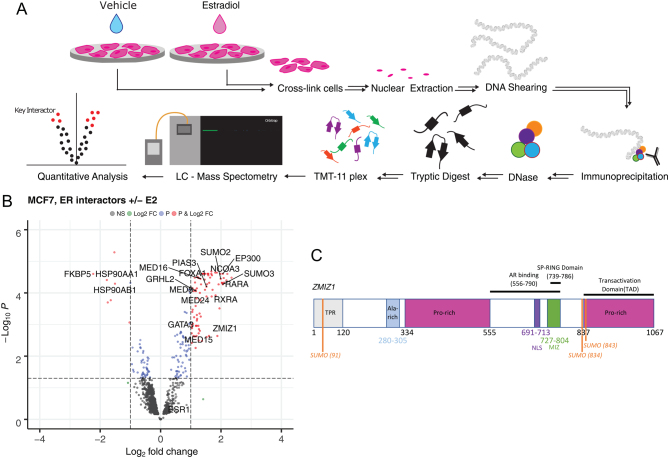
qPLEX-RIME of ER activation identifies novel co-factor ZMIZ1. (A) qPLEX-RIME enables the quantitative comparison of multiple conditions to identify key interactions between transcription factors on the chromatin. We stimulated ER-positive breast cancer cell lines with estradiol and undertook a comparative analysis against an unstimulated control to identified the changes in the ER interactome on activation. (B) ER interacting proteins identified by qPLEX-RIME in MCF7. Top ranking differential-associated proteins detected with *P<* 0.05 and LogFC *>*1 are highlighted in red. Gain of known co-factors GATA3 (Theodorou *et al.* 2013), RARA (Ross-Innes *et al.* 2010), EP300, GRHL2 (Holding *et al.* 2019) and NCOA3 were all detected along with the loss of HSP90 on binding of estradiol by the ER. Parts of the mediator complex (MED8, MED16 and MED24), along with pioneer factor FOXA1 were also detected (Supplementary Table 1). ZMIZ1 has not previously been reported to interact with the ER. (C) A schematic presentation of the ZMIZ1 protein. The AR binding region was previously identified by the interaction of a 556-790aa truncated mutant with an AR-GAL4-DBD fusion protein leading to activation of β-gal reporter gene. The C-terminal, proline-rich region of ZMIZ1, was identified as an intrinsic transactivation domain (TAD) (Sharma *et al.* 2003). TPR, tetratricopeptide repeat (Wang *et al.* 2018); NLS, nuclear localisation signal (Sharma *et al.* 2003). A full colour version of this figure is available at https://doi.org/10.1530/JME-23-0133.

We used qPLEX-RIME, a state-of-the-art method for quantifying protein–protein interactions within nuclear receptor complexes (Papachristou *et al.* 2018), to quantitatively monitor the changes in protein–protein interactions in the ER complex between 0 and 45 min in response to activating MCF7 cells (ER-positive cell line) with estradiol (Fig. 1A). The assay was repeated across four isogenic replicates.

Detected protein–ER interactions that were found to significantly change on stimulation with estradiol (*P<* 0.05) and had a two-fold change in protein intensity were reviewed for known biology. We found several previously identified ER co-factors including RARA, CBP, EP300, NRIP1 and GATA3. We also detected SUMO1-3 within the ER complex, most likely as a result of the covalent modification of the ER-α or another protein within the ER chromatin-bound complex, in agreement with previous ChIP-seq experiments (Traboulsi *et al.* 2018). In addition, we found novel putative ER interactions within the data (Fig. 1B and Supplementary Table 1).

The list of potential new ER co-factors includes ZMIZ1 (Fig. 1C), which had previously been identified as a co-activator of the androgen receptor (AR) (Li *et al.* 2011, Sharma *et al.* 2003). However, a key result of that study is that the effect was AR-specific in CV-1 cells. Transfection of ZMIZ1 into CV-1 cells was not able to enhance glucocorticoid receptor, progesterone receptor, ER, and vitamin D receptor-mediated transcription (Sharma *et al.* 2003). The AR specificity of the previous ZMIZ1 result has since been incorporated in the GeneCards and UniProt databases (UniProt Consortium 2019) and unpublished yeast data, discussed by Sharma *et al.* (2003), supports the literature that there is no interaction between ZMIZ1 and non-AR nuclear receptors. Given that our qPLEX-RIME data shows a significant enrichment of ZMIZ1 protein vs the control, was undertaken using a breast cancer cell line, and that we did not detect the presence of AR in our qPLEX-RIME data, this implies AR was not within the ER complex in the experimental conditions used. Therefore, we considered the breast cancer specific function of ZMIZ1 to be of key interest for follow-up studies.

### Proximity ligation assay confirms that the ZMIZ1 and ER proteins are within 40 nm in ER-positive cell lines

**Figure 2:**
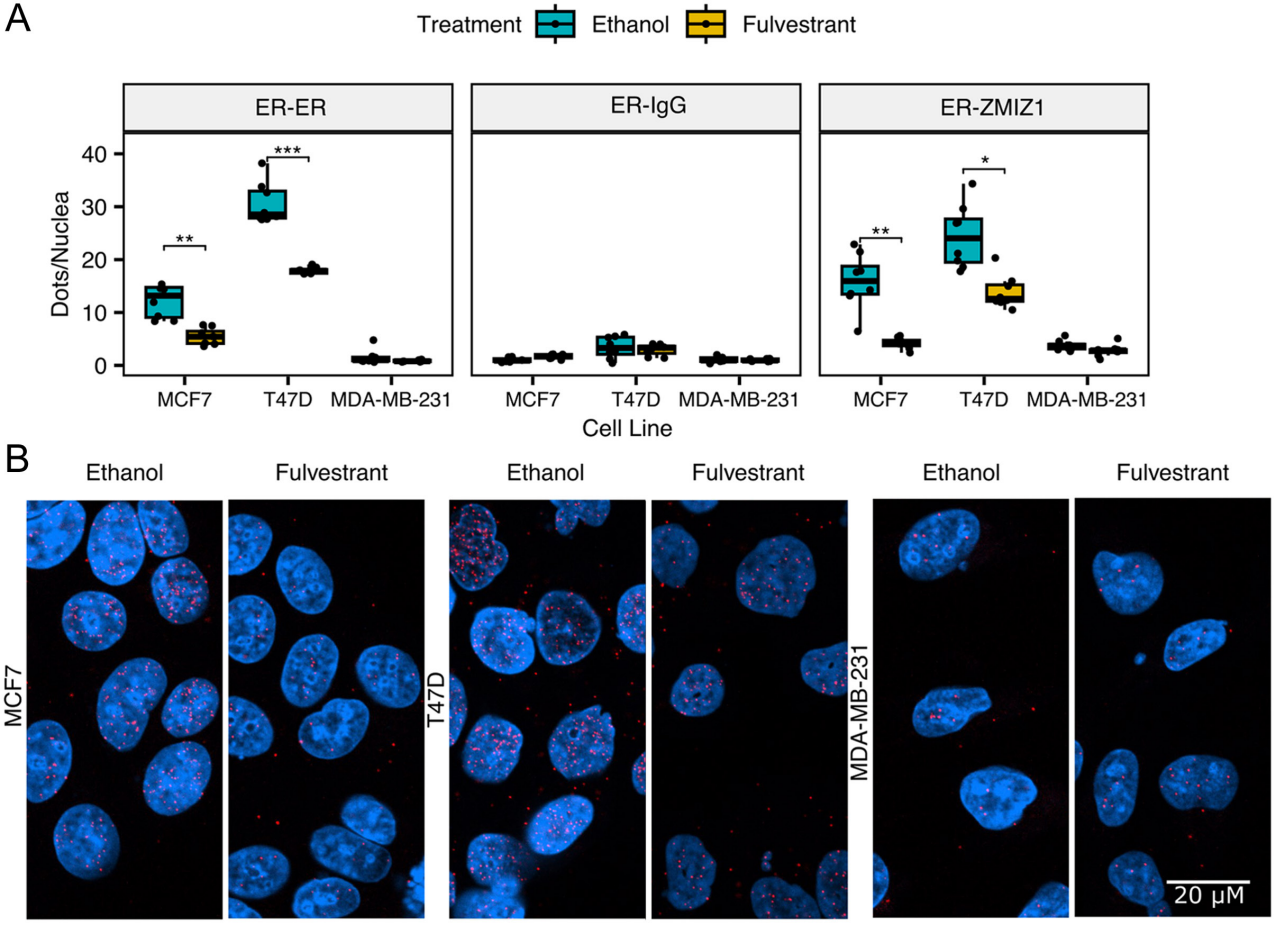
ZMIZ1 is within the ER transcriptional complex. (A) Cells were treated for 24 h with vehicle (ethanol) or with 100 nM fulvestrant (a selective ER degrader) before analysis by PLA. Dual antibody recognition PLA between ER (Ab3575) and either ER (SC8002), IgG or ZMIZ1 demonstrated a significant reduction in both ER and the ER-ZMIZ1 interaction on addition of fulvestrant in ER-positive cell lines, indicating that ER and ZMIZ1 are within the same protein complex. No significant effect on the PLA signal was detected in the ER-IgG negative control or in the ER-negative cell line MDA-MB-231 (Bonferroni adjusted *P*-value, ^*^<0.05, ^**^<0.01, ^***^<0.001). The error bars indicate s.d. (B) Illustrative images of the ER-ZMIZ1 PLA, taken at 630× magnification. DAPI stained cell nuclei are shown in blue and PLA signals are shown as red dots.

We applied PLA to validate our qPLEX-RIME results. PLA signal is specific to proteins that are within 40 nm (Alam 2022), and we successfully detected the proximity of ER and ZMIZ1 in both of the nuclei of two ER-positive cell lines (MCF7 and T47D). Our analysis of the ER-ZMIZ1 proximity in the ER-negative cell line, MDA-MB-231, demonstrated no significant signal over |our ER-IgG dual recognition PLA negative control, indicating the signal was specific to ER-positive cell lines. To confirm the proximity of ER-ZMIZ1 was dependent on ER protein, we treated the three cell lines with 100 nM fulvestrant, a Selective ER Degrader (SERD). On treatment, we measured a significant reduction (adjusted *P*-value = 0.0029 and 0.0101 for MCF7 and T47D, respectively) in the number of PLA signals in ER-positive cell lines for the ER-ZMIZ1 dual recognition PLA assays when compared to the vehicle control (Fig. 2A–B). To confirm the specificity of the signal to ER protein, we conducted an ER–ER PLA. The results showed a significant reduction in PLA signal in both MCF7 and T47D cells, confirming the specificity of our antibodies to the ER protein. Additionally, to verify if the PLA signal was also reliant on the ZMIZ1 protein, we carried out a final PLA control experiment assessing the impact of ZMIZ1 knockdown on the ER–ZMIZ1 signal (Supplementary Fig. 2). Knockdown of ZMIZ1 significantly reduced the ER–ZMIZ1 PLA signal in both ER-positive cell lines tested, supporting the dependency of the PLA signal on the ZMIZ1 protein. Images of PLA controls and siZMIZ1 knockdown are shown in Supplementary Figs 3–7. We attempted to establish the interaction by co-IP (Supplementary Fig. 8) under native conditions. While we did detect the interaction of ER and ZMIZ1 under native pull-down conditions in the T47D cell line, we did not in the MCF7 or MDA-MB-231 cell lines. The result could not be reproduced using an ER pull-down followed by ZMIZ1 blotting. Our results therefore confirmed the ER and ZMIZ1 are within 40 nm of each other, and therefore likely within the same transcriptional complex in breast cancer cell lines. However, the evidence of a direct protein–protein interaction was minimal, in line with previously reported results that found the protein–protein interaction to be AR specific (Sharma *et al.* 2003).

### ZMIZ1 knockdown delays response to E2 in ER regulated cell cycle-related genes

Since ZMIZ1 has a previously described role as a co-activator of AR and PLA confirmed it was within the ER complex, we hypothesised that knockdown of ZMIZ1 would also result in a reduced response to E2 at downstream targets of the ER.

To test our hypothesis, we set up a paired RNA-seq experiment treating MCF7 cells with either siZMIZ1 or siCTRL. In both cases, we undertook four isogenic replicates, measuring transcriptional levels at 3, 6, 12 and 24 h after stimulation with estradiol. The reduction in expression of ZMIZ1 by siRNA knockdown was found to be significant at all time points compared to the control (adjusted *P*-value *<*0.01, *n* = 4, Supplementary Fig. 9). No significant change in the expression of the ER was detected (Supplementary Fig. 9). Knockdown of ZMIZ1 protein was confirmed by western blot 48 h after siRNA transfection (Supplementary Fig. 10).

Between the siZMIZ1 and control condition, the largest number of differentially expressed genes occurred at 6 h after stimulation with E2 (30 h after initial knockdown). In contrast, by 24 h (48 h after knockdown of ZMIZ1), only 14 genes were detected as differentially expressed (Supplementary Fig. 11).

We employed gene set enrichment analysis (GSEA) (Subramanian *et al.* 2005) to assess the differential expression of estrogen responsive genes at the 6-h mark between siZMIZ1 and siCTRL groups. Our study initially identified three pertinent gene sets from existing literature relevant to the employed cell culture models: STEIN_ESR1_TARGETS (Stein *et al.* 2008), BHAT_ESR1_TARGETS_NOT_VIA_AKT1_UP (Bhat-Nakshatri *et al.* 2008) and WILLIAMS_ESR1_TARGETS_UP (Williams *et al.* 2008). The GSEA of our RNA-seq data 6 h post E2 stimulation in MCF7 cells with these gene sets gave mixed results. Specifically, STEIN_ESR1_TARGETS exhibited a non-significant decrease in expression (*P* = 0.07), BHAT_ESR1_TARGETS_NOT_VIA_AKT1_UP displayed a notable increase (*P* = 0.003) and WILLIAMS_ESR1_TARGETS_UP showed a significant decline in ER transcriptional activity (*P* = 0.047) (Supplementary Fig. 11). Upon examining the concurrence among the three gene sets, it was observed that the overlap is minimal with only three genes shared across all sets. This feature provided an explanation for our divergent results and led us to hypothesise that ZMIZ1 had a specific functional role with the ER that was not equally represented in all of the three gene sets.

Given the ER’s role in regulating cell cycle (JavanMoghadam *et al.* 2016) and our interest in cancer progression, we undertook GSEA against three MSigDB gene sets (Subramanian *et al.* 2005) focused on cell cycle: GO_CELL_CYCLE, KEGG_CELL_CYCLE and REACTOME_CELL_CYCLE. All three gene sets gave significant results (*P* = 2.3 × 10^–6^, 0.0001, and 3.5 × 10^–7^ respectively, Supplementary Fig. 11).

On the basis of these results, we hypothesised that ZMIZ1 regulated a subset of ER-regulated genes focused on cell cycle. To test our hypothesis, we undertook GSEA against the intersection of the GO_CELL_CYCLE, KEGG CELL_CYCLE and REACTOME_CELL_CYCLE gene sets with the previously tested ER-specific gene sets (gene lists provided in Supplementary Table 2). To confirm that the majority genes in each of the gene sets were estrogen responsive, we undertook differential expression analysis of all three cell cycle gene sets. In each case, the majority of gene transcripts in the gene sets significantly increased in abundance after stimulation with estrogen vs a vehicle control (Supplementary Fig. 12). The small number of transcripts that did not respond to estrogen were kept in the gene set for subsequent testing to ensure an unbiased approach to gene set creation.

Reanalysis of the siZMIZ1 knockdown data using the three new gene sets (GO cell cycle/ER Overlap, KEGG cell cycle/ER overlap and Reactome cell cycle/ER overlap) demonstrated that all three gene sets were significantly enriched in the MCF7 RNA-seq data (*P*= 0.0001, 0.0023, 0.0003, respectively, GSEA, Supplementary Fig. 13) with negative effect size. Overall, these results indicated a consistent reduction in the expression of these gene sets as a result of ZMIZ1 knockdown. Repeating the analysis in T47D cells also found all three gene sets to be significant (*P*-values = 0.0063, 0.032 and 0.0035, GSEA, Supplementary Fig. 13), in agreement with our results in MCF7 cells.

### ZMIZ1 and ER co-bind and co-regulate cell cycle regulator E2F2

**Figure 3:**
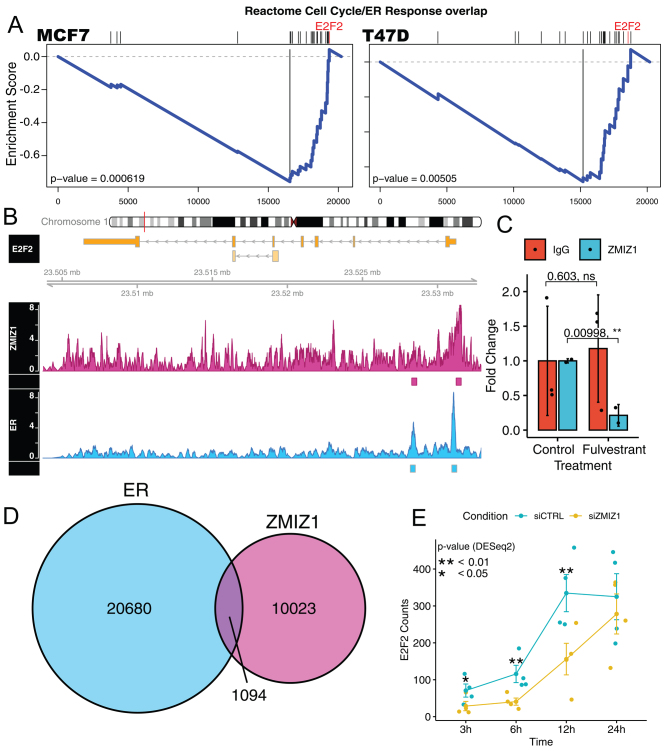
ZMIZ1 knockdown leads to a delay in the transcriptional response of estrogen regulated cell cycle genes. ER and ZMIZ1 both bind the E2F2 promoter. (A) GSEA of RNA-seq data generated 6 h after stimulation of cells by estrogen found significant depletion in our gene set created from the intersection of cell cycle and estrogen response genes in cells with ZMIZ1 knocked down. (B) Analysis of cell cycle genes for overlapping ER and ZMIZ1 binding sites identified a gene locus within the promoter of E2F2, a gene also found within our gene set. Coverage maps for ZMIZ1 and ER (ENCFF042TOP, ENCFF237WTX) showed a peak in the ChIP-seq signal for both factors at the promoter of the E2F2 gene, called and annotated by the ENCODE pipeline. A second peak within the first intron was also annotated in both ENCODE data sets, but the peak at the annotated site was not visually distinguishable from background in the ZMIZ1. (C) ChIP-qPCR validation of the ZMIZ1 binding site identified in the ENCODE ChIP-seq data confirmed significant loss of enrichment at E2F2 promoter DNA in the ZMIZ1 pulldown when MCF7 cells are treated with fulvestrant. Fulvestrant had no significant effect on the IgG control. (D) Overlap of ZMIZ1 binding in K562 cells (ENCFF881DAT) with ER binding in MCF7 (ENCFF138XTJ) downloaded from ENCODE showed that 10% of ZMIZ1 binding sizes overlapped with ER. (E) Reanalysis of the E2F2 transcript within our RNA-seq data showed significantly reduced expression at 3−12 h (*P<* 0.05). The *P*-value was calculated using DESeq2. A full colour version of this figure is available at https://doi.org/10.1530/JME-23-0133.

As ZMIZ1 and ER are both found in DNA-binding complexes, we aimed to establish if the proteins were bound in proximity of the same DNA elements that regulate cell cycle. We surmised that any sites we identified could indicate a potential cell cycle regulatory mechanism involving both ER and ZMIZ1 proteins. For ZMIZ1 binding, we used the ENCODE data reported in K562 cells, as no data for MCF7 cells was available, and for ER binding, we used data as reported by ENCODE in MCF7 cells (Luo *et al.* 2020). Overlaying these data demonstrated that nearly 10% of ZMIZ1, 1094 sites in total, were found to also bind ER (Fig. 3D) in support of our PLA results. Comparison of the overlap in ER and ZMIZ1-binding sites with our GSEA gene set identified E2F2 as being within both data sets (Fig. 3A). ENCODE reported two binding sites that passed their QC pipeline within the E2F2 gene loci for both ER and ZMIZ1, and both sites overlapped. Visual inspection of the ENCODE data showed clear binding of ER and ZMIZ1 at E2F2 promoter region. While the second binding site within the E2F2 intron was clearly bound by ER, the intronic ZMIZ1 ChIP-seq peak was at similar levels to background noise (Fig. 3B). To address concerns that the ENCODE ChIP-seq data was generated in K562 cells and shows poor enrichment over noise, we validated the ZMIZ1 binding of the E2F2 promoter in the MCF7 cell line. Our ChIP-qPCR results confirmed that the ENCODE ZMIZ1 ChIP-seq result was generalisable to our breast cancer model and that degrading ER with fulvestrant led to the loss of ZMIZ1 binding but had no effect on our IgG control ChIP (Fig. 3C). Further analyses of the RNA-seq data showed a reduction in E2F2 expression, with a maximum log_2_fold change of −1.6 at 3 h, and a log_2_-fold change of −1.4 between 6 and 12 h (Fig. 3E). The *P*-values for the siZMIZ1/siCTRL comparison of the E2F2 transcript as calculated by DESeq2 at the 3-, 6-, 12-, and 24-h time points were *P* = 0.01, 0.007, 0.007 and 0.5, respectively. Overall, these results indicate a delayed estrogenic expression of the E2F2 transcription factor as a result of the loss of the ZMIZ1 at the E2F2 promoter. We also noted, E2F2 was previously reported as critical for ER-positive breast tumour biology and disease progression (Nguyen-Vu *et al.* 2013).

### ZMIZ1 knockdown reduces proliferation of breast cancer cell lines

On the basis of these results, we tested the hypothesis that knockdown of the ZMIZ1 protein would result in reduced proliferation of ER-positive cancer cell line models.

**Figure 4:**
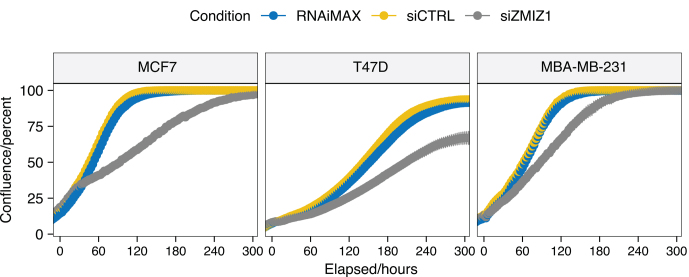
ZMIZ1 knockdown reduces ER-positive breast cancer cell line proliferation. Knockdown of ZMIZ1 in three cell lines (T47D, MCF7 and MDA-MB-231) all showed reduced proliferation. The effect was largest in the ER-positive cell lines, T47D and MCF7. A full colour version of this figure is available at https://doi.org/10.1530/JME-23-0133.

Analysis of two ER-positive (MCF7 and T47D) and one triple negative breast cancer (TNBC) model (MDA-MB-231) showed that knockdown of ZMIZ1 reduced cell proliferation in all three cell lines (Fig. 4) (ANOVA, *P<* 0.001).

A multi-way ANOVA was performed to analyse the effect of cell line and elapsed time on the ratio of cell confluence between siZMIZ1 and siCTRL conditions at each time point (herein called the confluence ratio). Both cell line and elapsed time were found to have a significant effect (*P<* 0.001) on the confluence ratio. The two independent variables, time and cell line, were also found to have a significant interaction (*P<* 0.001). These results fit with the visual inspection of the data that the effect of ZMIZ1 knockdown varies over the experiment time course, and that it is different between cell lines. Follow-up pairwise *t*-test comparison of the confluence ratio confirmed a significant difference in confluence ratio for MCF7 and MDA-MB-231 (adjusted *P*-value = 7.2 × 10^–7^, Benjamini–Hochberg correction) and T47D and MDA-MB-231 (adjusted *P<* 0.001, Benjamini–Hochberg). *P*-values smaller than R’s precision are reported as *P<* 0.001 as they cannot be stated exactly. We also noted that the T47D growth reached a maximum at a reduced confluence compared to the control experiments, which was not seen in either of the other two cell lines.

The significant difference in confluence ratio (i.e. effect size of the ZMIZ1 knockdown on cell growth between the ER+ and TNBC cell lines) evidences that targeting ZMIZ1 has a greater effect on the growth of T47D and MCF7 cell lines (ER-positive) than on the MDA-MB-231 cell line (TNBC).

### High ZMIZ1 expression correlates with low survival in ER-positive patients

To explore if the role of ZMIZ1 activity held clinical importance, we investigated if ZMIZ1 expression was a predictor of patient survival in the context of both ER-positive and ER-negative breast tumours using KMplot (Lanczky *et al.* 2016).

**Figure 5:**
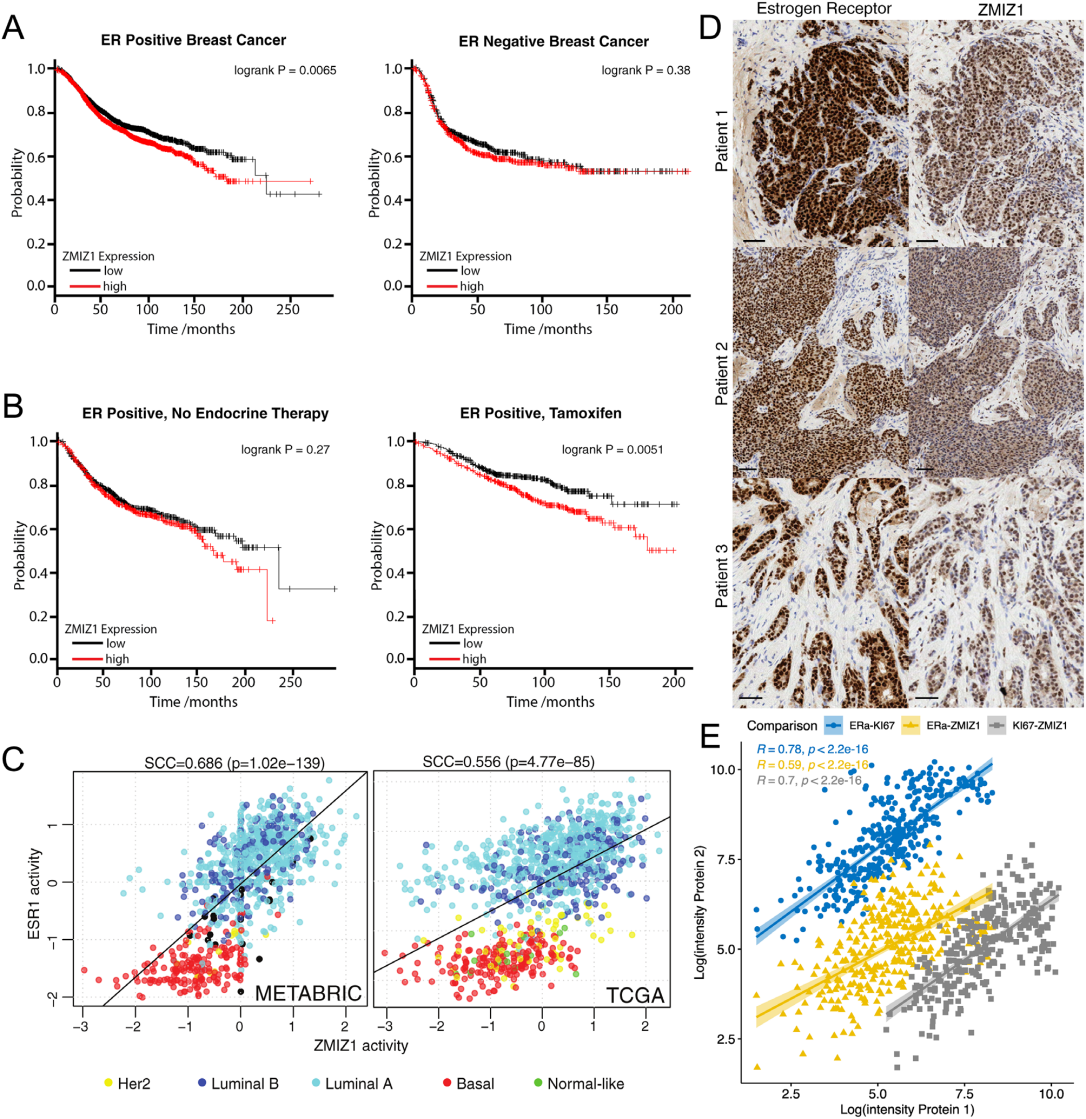
High ZMIZ1 expression correlates with low survival in ER-positive patients, ZMIZ1 and ER transcriptional activities correlate in patient samples, and ZMIZ1 and ER are co-localised in patient samples. (A) Stratifying patients of ER-positive (left) breast cancer based on ZMIZ1 expression shows that high levels of ZMIZ1 results in a significant reduction in patient survival. The effect is not seen with ER-negative (right) breast cancer, implying an ER-positive specific function for ZMIZ1 in breast cancer. (B) Stratifying ER-positive breast cancer based on treatment, either no endocrine therapy or tamoxifen only, shows that high levels of ZMIZ1 results in a significant reduction in patient survival for patients who receive tamoxifen. The effect is not seen for the ‘no endocrine therapy’ patients (left), further supporting an interaction between ZMIZ1 with the ER signalling pathway in breast cancer. (C) Transcriptional activity of ESR1 and ZMIZ1, as measured by the VIPER algorithm, correlate in both the METABRIC and TCGA cohort, thus confirming both transcription factors are transcriptionally active within ER-positive tumours and potentially function together in ER-positive tumours. (D) Consecutive slides derived from biopsy samples of three patients with ER-positive breast cancer were stained by IHC for both the estrogen receptor-α and ZMIZ1. In all three patients, the localisation of staining for the proteins was specific to the tumour cells and the nucleus. In contrast, the stromal cells show little or no expression of either protein. Scale: 50 µm. (E) Correlation of protein abundance for ZMIZ1, ER and KI67 in breast cancer tumours in the TCGA cohort (from PXD024322). ZMIZ1 correlates significantly with KI67 protein expression, a marker of proliferation.

Stratifying ER-positive patients (ER status by array) by median ZMIZ1 expression showed that ER-positive patients with higher levels of ZMIZ1 expression had significantly poorer outcome (*P* = 0.0065, logrank test, hazard ratio (HR) = 1.18 (1.05–1.33)). In contrast, the same analysis of ER-negative patients demonstrated no significant difference in patient outcome (*P* = 0.38, logrank test, Fig. 5A). Survival differences were also confirmed in METABRIC (Curtis *et al.* 2012) and TCGA (Weinstein *et al.* 2013) ER-positive breast cancer cohorts (Supplementary Fig. 14).

Splitting the ER-positive patient cohort into those who received no endocrine therapy and those on tamoxifen only (Fig. 5B) showed that ZMIZ1 expression had no significant effect on the survival of those who received no endocrine therapy (*P* = 0.27, logrank test, HR = 1.12 (0.92–1.37)). Patients who did receive tamoxifen and who expressed greater than median levels of ZMIZ1 saw a significant reduction in recurrent-free survival (*P* = 0.0051, logrank test, HR = 1.52 (1.13–2.05)).

These results were consistent with the ZMIZ1 functioning as part of the ER protein complex and that ZMIZ1 plays a role in disease progression in ER-positive breast cancer.

### ZMIZ1 and ER activities correlate in patient samples

If ZMIZ1 is a component of the ER transcriptional complex, we would expect its activity to be higher in ER-positive than in ER-negative breast cancer. To test this hypothesis, we used two large gene expression collections, TCGA (Weinstein *et al.* 2013) and METABRIC (Curtis *et al.* 2012), to assess co-expression of ER and ZMIZ1 in a patient setting. In both data sets, ZMIZ1 had significantly increased expression in luminal over basal sub-types (TCGA: *P<* 2.22 × 10^−16^ for both luminal A and B vs basal, Wilcoxon test, *n* = 572, 223 and 173 respectively; METABRIC: *P* = 3.1 × 10^-9^ and *P* = 1.6 × 10^−9^ for luminal A and B vs basal, respectively, Wilcoxon test, *n* = 590, 203, 168 respectively) (Supplementary Fig. 15).

To address if both ZMIZ1 and ER proteins were transcriptionally active within patient samples, we then generated regulatory network models for both the TCGA and METABRIC data sets using ARACNe-AP (Lachmann *et al.* 2016). Using these networks, we applied the VIPER algorithm (Alvarez *et al.* 2016) to calculate the activity of ER and ZMIZ1 proteins in both patient sample data sets (Fig. 5C). For both TCGA and METABRIC, we saw a significant correlation (*P* = 4.77 × 10^−85^ and*P* = 1.02 × 10^−139^ respectively) between the activity of the two transcription factors, with greatest activity of both networks in the luminal sub-type.

### Immunohistochemistry of ZMIZ1 and ER demonstrates both proteins locate in the same regions of patient tumours

To further validate the link between ER and ZMIZ1, we checked if ZMIZ1 expression was found in the ER-positive tumour cells in clinical material. Visual inspection of ER-positive breast cancer tumours from all three patients analysed showed strong nuclear staining of both proteins in adjacent sections. Comparison of the localisation of staining between ER and ZMIZ1 demonstrated that both proteins were found within the nucleus of epithelial cells. Neither ER nor ZMIZ1 was detected in the infiltrating cells. Further, the distribution of ER and ZMIZ1 staining correlated, implicating that ER and ZMIZ1 are expressed within the same cells of the patient tumours (Fig. 5D).

### ZMIZ1 and marker of proliferation KI67 protein levels significantly correlate in patient tumours

To establish if ZMIZ1 protein abundance correlated with cell proliferation (KI67) rate, we undertook the analysis of the published TCGA quantitative proteomic data (PXD024322) (Asleh *et al.* 2022). All of ZMIZ1, KI67 and ER protein abundances correlated strongly (*P<* 0.001, Pearson’s) (Fig. 5E) with each other. The associated R-value showed a higher correlation between ZMIZ1 and KI67 (0.70) than between ER and ZMIZ1 (0.59). The highest correlation was between ER and KI67 (0.78). Partial correlation (Pearson’s) was applied to confirm if ZMIZ1 protein expression had an effect on KI67 protein independent of ER. The correlation between ZMIZ1 and KI67 was significant at 0.46 (*P<* 4.9 × 10^−21^) when controlling for ER protein abundance. For comparison, the correlation of ER to KI67 controlling for ZMIZ1 protein abundance was 0.63 (*P<* 1.7 × 10^−42^). Overall, these results confirmed the ZMIZ1 expression correlated with marker of proliferation KI67 significantly and in addition to the ZMIZ1 protein abundance correlating with ER protein expression.

## Discussion

All previous evidence supported the conclusion that ZMIZ1 was an AR-specific co-activator (Sharma *et al.* 2003). Results in the cited study demonstrated that AR, ER and other steroid hormone receptors could all activate transcription when presented with their respective ligands in the cell line models used. However, only the activity of AR was linked to the levels of ZMIZ1 within the cell. Therefore, on detection of ZMIZ1 within the ER complex in the absence of AR, we hypothesised that the absence of evidence for an ER-ZMIZ1 interaction until this point may be as a result of the cell line models used in previous research.

Undertaking analysis in ER-positive breast cancer cell line models, we demonstrated a significant change in the ER-mediated transcriptional response on ZMIZ1 knockdown. Further, we were able to show that within patient samples where ZMIZ1, ER and KI67 protein expressions correlated, ZMIZ1 expression is predictive of survival, and the proteins are both localised within the nuclei of patient tumour cells. Combined, these results provide good evidence of an important role for ZMIZ1 in ER-positive breast cancer.

Previously, it has been shown that ZMIZ1 has a very strong transactivation domain (TAD) (Sharma *et al.* 2003) and our findings do not rule out this mechanism. However, in stark contrast to the AR-ZMIZ1 interaction, our research, along with that of other investigators, yielded no definitive evidence of a direct protein-protein interaction through co-immunoprecipitation. Instead, we have shown the two proteins are in close proximity via PLA and both proteins bind the E2F2 promoter by ChIP-qPCR. An explanation for the discrepancy in our results is that PLA detects proteins within the complex through space, and qPLEX-RIME uses cross-linking to both DNA and protein to capture transient interactions, while native Co-IP is more dependent on the protein−protein affinities within the protein complex. These observations, and the reported results that ZMIZ1 cannot co-activate ER in the original AR-ZMIZ1 interaction studies, suggest that the ER−ZMIZ1 interaction in breast cancer may be via a different mechanism to that in the prostate. One potential alternative is ZMIZ1 may activate the ER by promoting the SUMOylation of either the ER or its co-factors via its SP-RING domain (Li *et al.* 2011). A similar role has been seen for the AR, increasing SUMOylation by about 40% (Sharma *et al.* 2003), and our own qPLEX-RIME analysis of the ER shows an increase in the identified SUMO protein modifications on stimulation estradiol.

Taken together, we therefore propose that ZMIZ1 functions as part of the estrogen response by being in close proximity to, or in, the ER complex, thus enabling the efficient expression of cycle cell-related genes in response to estrogen due to a convergence of ER and ZMIZ1 activity at the E2F2 promoter in breast cancer. These findings imply that ZMIZ1 holds a previously undescribed and important role for tumour progression.

## Supplementary Materials

Table S1 – Summarised qPLEX-RIME results for differentially bound proteins detected with an adjusted p-value > 0.05. Full data is available in the R-package linked from the main text.

Table S2 – Estrogen Responsive Cell Cycle Gene sets.

Supplementary Figures

## Declaration of interests

FM is a founder, director, and shareholder of Tailor Bio. FM is a paid consultant for the Alan Turing Institute. AC started part-time employment with Tailor Bio during the completion of the manuscript. MG became CEO and co-founder at Cyted during completion of the manuscript.

## Funding

This work was funded by CRUK core grant C14303/A17197 and A19274 (to FM), The Alan Turing Institutehttp://dx.doi.org/10.13039/100012338 under the EPSRC grant EP/N510129/129/1 as a Turing Fellowship and BBSRC grant BB/V000071/1 (to ANH), and Royal Societyhttp://dx.doi.org/10.13039/501100000288 Research Grant RGS/R2/202120 (to KSB and ANH).

## Ethical approval

All samples analysed by IHC were collected as part of the PIONEER trial, REC number 17/NE/0113.

## Data and code availability

All RNA-seq data have been deposited in the GEO database with the accession number GSE133381. The code and data to generate the figures in this paper are available as an R-package (https://doi.org/10.5281/zenodo.10973173) from https://github.com/andrewholding/ZMIZ1.

## Author contribution statement

WZ, JS, AG, RB, AEC, LB, SFR and ANH undertook the experimentation. SK provided patient material. AG and SK generated ZMIZ1 stained images of patient samples. JS. AG, RB, KK, AS, ME, FMG, SFR and ANH analysed the data. MG analysed the ZMIZ1 stained images of patient samples. FMG undertook network analysis of TGCA and METABRIC data sets. AG, KSB and ANH designed the experiments. WZ, AG, SFR, ANH and FM wrote the manuscript. All authors were involved in the proofing and editing of drafts. We would like to acknowledge support of the Imaging and Cytometry group in the Technology Facilities at The University of York and the contribution from the CRUK Genomics, Proteomics, Histopathology/ISH, and Bioinformatics core facilities in supporting this work.
