# Supplementary material for: ZMIZ1 enhances ERα-dependent expression of E2F2 in breast cancer
Source: J Mol Endocrinol. 2024 Apr 25;73(1):e230133. doi: 10.1530/JME-23-0133 (PMC11103680; doi:10.1530/JME-23-0133)
Supplement: Table S1 – Summarised qPLEX-RIME results for differentially bound proteins detected with an adjusted p-value > 0.05. Full data is available in the R-package linked from the main text. [file supplementary_table_1.pdf]

Table S1 – Summarised qPLEX-RIME results for differentially bound proteins detected with an adjusted p-value > 0.05. Full data is available in the R-package linked from the main text.

	Accession	Protein Symbol	Fold Change	P-value	Adjusted P-Value
1	Q9C091	GREB1L	-1.53	3.1E-09	5.2E-06
2	Q15532	SS18	1.68	2.6E-08	2.1E-05
3	Q8TAQ2	SMARCC2	1.86	5.4E-08	2.3E-05
4	Q9Y2G9	SBNO2	1.81	5.7E-08	2.3E-05
5	P55317	FOXA1	1.58	8.4E-08	2.5E-05
6	P11474	ESRRA	1.45	1.5E-07	2.5E-05
7	P63279	UBE2I	1.48	1.6E-07	2.5E-05
8	Q9Y4B4	RAD54L2	2.21	1.7E-07	2.5E-05
9	Q13451	FKBP5	-2.23	1.7E-07	2.5E-05
10	Q9Y6Q9	NCOA3	1.98	1.7E-07	2.5E-05
11	Q09472	EP300	2.06	1.9E-07	2.5E-05
12	O75925	PIAS1	1.38	1.9E-07	2.5E-05
13	Q15596	NCOA2	1.68	2.1E-07	2.5E-05
14	P40424	PBX1	1.56	2.1E-07	2.5E-05
15	O43474	KLF4	1.43	2.3E-07	2.5E-05
16	O75177	SS18L1	1.39	2.4E-07	2.5E-05
17	O43791	SPOP	1.7	2.9E-07	2.8E-05
18	Q86Z02	HIPK1	1.58	3.1E-07	2.8E-05
19	P51532	SMARCA4	1.22	3.6E-07	3.1E-05
20	P28702	RXRB	1.64	4.1E-07	3.3E-05
21	Q13485	SMAD4	2.35	4.3E-07	3.3E-05
22	O95365	ZBTB7A	1.13	4.5E-07	3.3E-05
23	O14686	KMT2D	1.25	4.8E-07	3.4E-05
24	Q92793	CREBBP	2.09	5E-07	3.4E-05
25	O14497	ARID1A	1.26	5.1E-07	3.4E-05
26	Q9Y2X0	MED16	1.32	5.7E-07	3.6E-05
27	P61956	SUMO2	1.99	5.9E-07	3.6E-05
28	Q8TE85	GRHL3	1.46	6.4E-07	3.8E-05
29	P07900	HSP90AA1	-1.78	6.9E-07	3.9E-05
30	Q13887	KLF5	2.18	7.3E-07	4E-05
31	Q86U70	LDB1	1.14	9E-07	4.7E-05
32	P48552	NRIP1	2.1	9.1E-07	4.7E-05
33	Q6IQ16	SPOPL	1.61	9.8E-07	4.8E-05
34	O15164	TRIM24	2.03	1E-06	4.8E-05
35	P31689	DNAJA1	-1	1E-06	4.8E-05
36	P10276	RARA	2.04	1.1E-06	4.9E-05
37	P08238	HSP90AB1	-1.51	1.2E-06	5.1E-05
38	Q15788	NCOA1	1.54	1.2E-06	5.3E-05
39	Q6EEV6	SUMO4	1.92	1.3E-06	5.4E-05

40	Q9BRQ0	PYGO2	1.34	1.3E-06	5.4E-05
41	P55854	SUMO3	2.06	1.3E-06	5.4E-05
42	Q9Y6X2	PIAS3	1.52	1.6E-06	6.4E-05
43	Q6ZW49	PAXIP1	1.06	1.9E-06	7.2E-05
44	O00712	NFIB	1.2	1.9E-06	7.2E-05
45	Q8NEZ4	KMT2C	0.94	2.2E-06	7.9E-05
46	P31276	HOXC13	1.47	2.2E-06	8E-05
47	Q92754	TFAP2C	1.72	2.3E-06	8E-05
48	Q5JPI3	C3orf38	1	2.3E-06	8E-05
49	Q96G25	MED8	1.15	2.4E-06	8.1E-05
50	Q6ISB3	GRHL2	1.17	2.7E-06	8.8E-05
51	Q01196	RUNX1	1.1	2.7E-06	8.8E-05
52	Q92925	SMARCD2	0.97	3.2E-06	1E-04
53	O75448	MED24	1.3	3.3E-06	1E-04
54	Q9UPN9	TRIM33	1.82	4E-06	0.00012
55	O15550	KDM6A	1.52	4.3E-06	0.00013
56	P19793	RXRA	1.69	4.4E-06	0.00013
57	P46937	YAP1	1.19	5E-06	0.00015
58	Q9NWF9	RNF216	1.31	5.9E-06	0.00017
59	Q02790	FKBP4	-1.65	6.1E-06	0.00017
60	Q4ZG55	GREB1	-1.75	7.2E-06	2E-04
61	O75081	CBFA2T3	1.16	9.5E-06	0.00026
62	O60479	DLX3	1.95	1.2E-05	0.00031
63	P84022	SMAD3	1.06	1.3E-05	0.00034
64	P40763	STAT3	0.8	1.6E-05	4E-04
65	Q9UKL0	RCOR1	1.05	1.6E-05	4E-04
66	Q969G3	SMARCE1	0.86	1.7E-05	0.00043
67	Q9UBL3	ASH2L	1.29	1.8E-05	0.00045
68	P10588	NR2F6	1.2	2.1E-05	5E-04
69	Q9UER7	DAXX	1.33	2.3E-05	0.00056
70	Q86UU0	BCL9L	1.11	2.5E-05	0.00059
71	Q13950	RUNX2	1.21	2.9E-05	0.00066
72	Q5TC79	ZBTB37	1.2	3.3E-05	0.00075
73	O60884	DNAJA2	-1.03	3.8E-05	0.00086
74	Q9NZI5	GRHL1	0.99	3.9E-05	0.00086
75	P17275	JUNB	1.07	4.3E-05	0.00095
76	Q7Z5J4	RAI1	0.95	4.4E-05	0.00095
77	Q9UIU6	SIX4	1.25	5.3E-05	0.0011
78	Q6ZRI6	C15orf39	1.04	5.3E-05	0.0011
79	Q92785	DPF2	1.19	5.4E-05	0.0011
80	P23771	GATA3	1.17	5.5E-05	0.0011
81	P24468	NR2F2	1.04	6.6E-05	0.0013
82	Q9ULJ6	ZMIZ1	1.27	7.1E-05	0.0014

**83**	Q9UN79	SOX13	1.3	8.2E-05	0.0016
**84**	Q86X55	CARM1	0.67	8.7E-05	0.0017
**85**	O60341	KDM1A	0.68	9.4E-05	0.0018
**86**	P78413	IRX4	1.03	1E-04	0.0019
**87**	P39880	CUX1	0.65	1E-04	0.0019
**88**	Q96RN5	MED15	1.26	1E-04	0.0019
**89**	P35869	AHR	0.55	0.00012	0.0021
**90**	Q14190	SIM2	0.74	0.00012	0.0023
**91**	Q92908	GATA6	1.09	0.00013	0.0023
**92**	Q92973	TNPO1	0.48	0.00013	0.0023
**93**	Q9UBW7	ZMYM2	1.88	0.00013	0.0023
**94**	Q8IX15	HOMEZ	1.6	0.00013	0.0023
**95**	Q8N2W9	PIAS4	1.19	0.00013	0.0023
**96**	Q9C005	DPY30	1.13	0.00014	0.0024
**97**	Q6PJG2	ELMSAN1	0.75	0.00015	0.0025
**98**	P13631	RARG	1.52	0.00015	0.0025
**99**	Q9Y618	NCOR2	0.77	0.00015	0.0026
**100**	Q5HY92	FIGN	0.94	0.00017	0.0028
**101**	Q9UNE7	STUB1	-0.67	0.00019	0.0031
**102**	P18847	ATF3	1.26	2E-04	0.0032
**103**	Q14149	MORC3	1.37	0.00021	0.0033
**104**	O60885	BRD4	0.85	0.00022	0.0034
**105**	Q14686	NCOA6	0.57	0.00022	0.0035
**106**	Q04726	TLE3	0.73	0.00023	0.0036
**107**	P17535	JUND	1.03	0.00025	0.0038
**108**	P10644	PRKAR1A	-0.69	0.00028	0.0042
**109**	Q13547	HDAC1	0.66	0.00028	0.0042
**110**	Q15291	RBBP5	0.73	0.00028	0.0042
**111**	O15294	OGT	0.93	3E-04	0.0045
**112**	Q8IX07	ZFPM1	0.84	0.00031	0.0046
**113**	Q8IU60	DCP2	-0.67	0.00037	0.0053
**114**	Q8NFH4	NUP37	0.66	0.00037	0.0054
**115**	Q15185	PTGES3	-0.93	0.00038	0.0054
**116**	Q9P2K3	RCOR3	1.16	0.00039	0.0055
**117**	Q9BTE3	MCMBP	-0.66	4E-04	0.0056
**118**	Q08117	AES	0.94	0.00042	0.0058
**119**	Q9Y2X9	ZNF281	0.79	0.00048	0.0066
**120**	Q9HBE1	PATZ1	0.89	0.00048	0.0066
**121**	P23588	EIF4B	-0.57	5E-04	0.0069
**122**	P31948	STIP1	-0.76	0.00052	0.0071
**123**	P05412	JUN	0.86	0.00053	0.0071
**124**	P60842	EIF4A1	-0.58	0.00059	0.0079
**125**	Q9UDV7	ZNF282	0.84	0.00069	0.0091

**126**	Q8WVV4	POF1B	0.52	0.00075	0.0098
**127**	O15417	TNRC18	0.66	8E-04	0.01
**128**	Q96F44	TRIM11	0.86	0.00081	0.01
**129**	O14980	XPO1	0.45	0.00084	0.011
**130**	P20073	ANXA7	0.39	0.00087	0.011
**131**	P52948	NUP98	-0.43	0.00088	0.011
**132**	P06730	EIF4E	-0.4	0.00093	0.012
**133**	Q49A26	GLYR1	-0.58	0.001	0.013
**134**	Q13363	CTBP1	0.57	0.001	0.013
**135**	P16220	CREB1	0.95	0.0011	0.013
**136**	Q92614	MYO18A	-0.63	0.0011	0.014
**137**	P24928	POLR2A	-0.43	0.0012	0.014
**138**	Q5TGY3	AHDC1	0.6	0.0012	0.015
**139**	P35580	MYH10	-0.63	0.0013	0.016
**140**	O75362	ZNF217	0.73	0.0013	0.016
**141**	Q53GS9	USP39	-0.54	0.0013	0.016
**142**	Q9BV38	WDR18	-0.44	0.0015	0.018
**143**	O75665	OFD1	-0.95	0.0016	0.018
**144**	O15014	ZNF609	0.69	0.0018	0.021
**145**	Q9UEG4	ZNF629	0.58	0.0019	0.022
**146**	Q14240	EIF4A2	-0.47	0.0021	0.023
**147**	O76074	PDE5A	-0.56	0.0021	0.023
**148**	Q6UUV7	CRTC3	0.81	0.0021	0.024
**149**	P27824	CANX	-0.45	0.0022	0.025
**150**	Q15121	PEA15	-0.44	0.0024	0.026
**151**	Q9Y3C6	PPIL1	0.35	0.0025	0.027
**152**	Q6ZPD9	DPY19L3	-0.91	0.0025	0.027
**153**	Q15056	EIF4H	-0.44	0.0025	0.027
**154**	Q01664	TFAP4	0.56	0.0028	0.03
**155**	P05549	TFAP2A	0.83	0.0028	0.03
**156**	Q9H5N1	RABEP2	-0.4	0.0028	0.03
**157**	Q9NYL9	TMOD3	0.53	0.0029	0.03
**158**	Q86YP4	GATAD2A	0.52	0.0029	0.03
**159**	Q13951	CBFB	0.61	0.003	0.031
**160**	Q13464	ROCK1	-0.89	0.0031	0.031
**161**	Q08379	GOLGA2	-0.38	0.0031	0.031
**162**	Q9H910	JPT2	-0.58	0.0032	0.033
**163**	O15327	INPP4B	-0.43	0.0032	0.033
**164**	P78545	ELF3	0.75	0.0033	0.033
**165**	Q9HB71	CACYBP	-0.76	0.0033	0.033
**166**	Q14687	GSE1	0.55	0.0036	0.036
**167**	P04040	CAT	-0.6	0.0037	0.036
**168**	Q96KP4	CNDP2	-0.57	0.0037	0.037

**169**	Q9NPI6	DCP1A	-0.44	0.0039	0.038
**170**	O95758	PTBP3	-0.53	0.0039	0.038
**171**	Q9H334	FOXP1	0.67	0.0041	0.04
**172**	A6NKD9	CCDC85C	-0.45	0.0042	0.04
**173**	Q93062	RBPMS	-0.62	0.0042	0.04
**174**	Q13207	TBX2	0.53	0.0043	0.041
**175**	Q99594	TEAD3	0.66	0.0043	0.041
**176**	Q9BSF8	BTBD10	-0.51	0.0044	0.042
**177**	Q03112	MECOM	0.87	0.0047	0.044
**178**	O96019	ACTL6A	0.49	0.0048	0.044
**179**	Q13227	GPS2	0.6	0.0049	0.045
**180**	Q7Z478	DHX29	-0.5	0.0051	0.046
**181**	Q9BXW6	OSBPL1A	-0.58	0.0051	0.046
**182**	O95793	STAU1	-0.35	0.0052	0.047
**183**	Q9UQR1	ZNF148	0.57	0.0054	0.048
**184**	Q16543	CDC37	-0.65	0.0054	0.048
