# Supplementary material for: ZMIZ1 enhances ERα-dependent expression of E2F2 in breast cancer
Source: J Mol Endocrinol. 2024 Apr 25;73(1):e230133. doi: 10.1530/JME-23-0133 (PMC11103680; doi:10.1530/JME-23-0133)
Supplement: Table S2 – Estrogen Responsive Cell Cycle Gene sets. [file supplementary_table_2.pdf]

Table S2 – Estrogen Responsive Cell Cycle Gene sets.

	React Cell Cycle / Estrogen Responsive Overlap	GO Cell Cycle / Estrogen Responsive Overlap	Kegg Cell Cycle / Estrogen Responsive Overlap
	**E2F2**	CEP55	MCM4
	FEN1	CCNA2	CCND1
	AHCTF1	MCM4	MCM6
	NSL1	THBS1	MCM5
	MCM2	GINS2	CCNA2
	MCM4	SLC2A8	BUB1
	MCM5	KLK10	MCM2
	MCM6	E2F6	CDC25A
	PCNA	TUBA4A	CDC6
	GINS2	CCND1	CDC20
	POLE2	NOLC1	SKP2
	ZWILCH	RRM1	PCNA
	MCM10	MCM10	**E2F2**
	CCND1	SKP2	CCNE2
	RFC4	MCM5	
	SKP2	MCM2	
	BUB1	NCAPG2	
	TUBA4A	XRCC3	
	CENPU	DYNLT3	
	CCNA2	AUNIP	
	CCNE2	WDR62	
	CDC6	CDC6	
	CDC20	BUB1	
	CDC25A	PRR5	
		CDC20	
		SMC4	
		**E2F2**	
		DTL	
		CCNE2	
		CENPU	
		UHRF1	
		PCNA	

		ZWILCH	
		SIAH2	
		NSL1	
		CDC25A	
		FAM83D	
		MCM6	
		AHCTF1	
		POLE2	
		TPD52L1	
