# Supplementary material for: ZMIZ1 enhances ERα-dependent expression of E2F2 in breast cancer
Source: J Mol Endocrinol. 2024 Apr 25;73(1):e230133. doi: 10.1530/JME-23-0133 (PMC11103680; doi:10.1530/JME-23-0133)
Supplement: Supplementary Figures [file supplementary_figures.pdf]

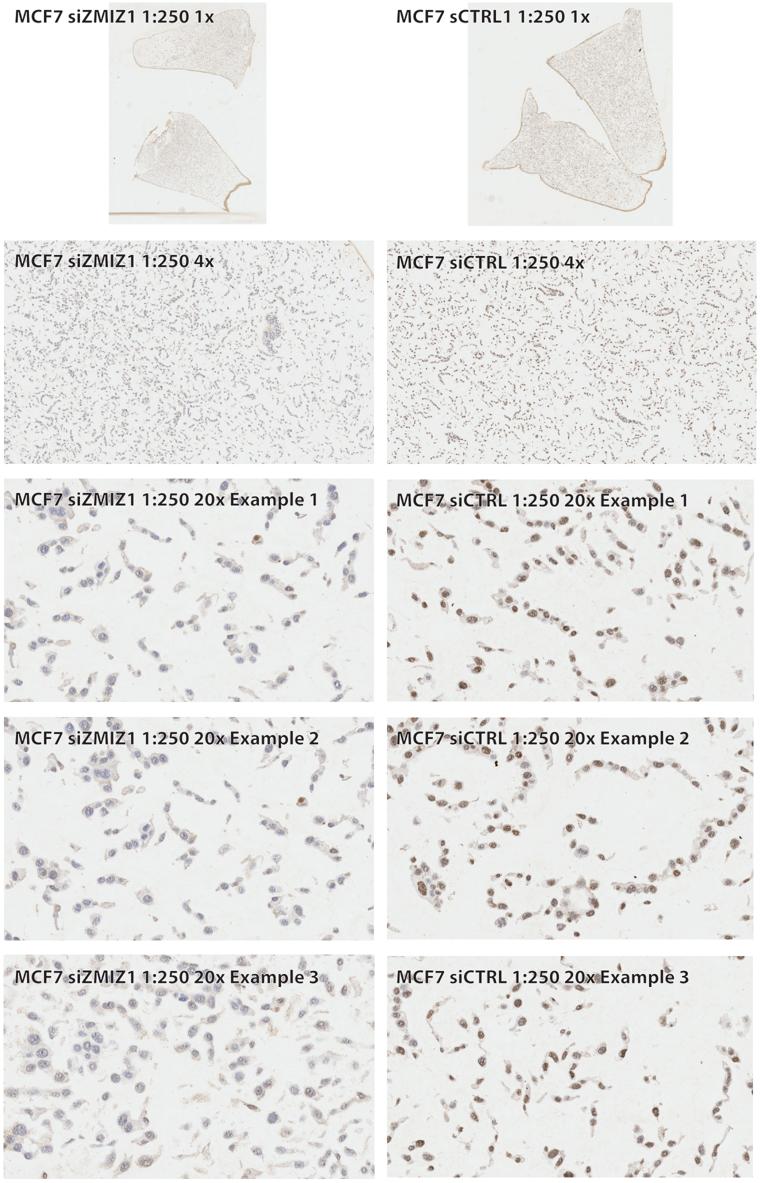

Figure S1: **ZMIZ1 antibody validation of IHC**. Formalin fixed pellets of MCF7 cells were processed as described in the methods section. The antibody (1:250 dilution) used for the images shown was purchased from R&D Systems (AF8107) and used for all subsequent samples. Nuclear staining of ZMIZ1 was visibly reduced in the siZMIZ1 condition.

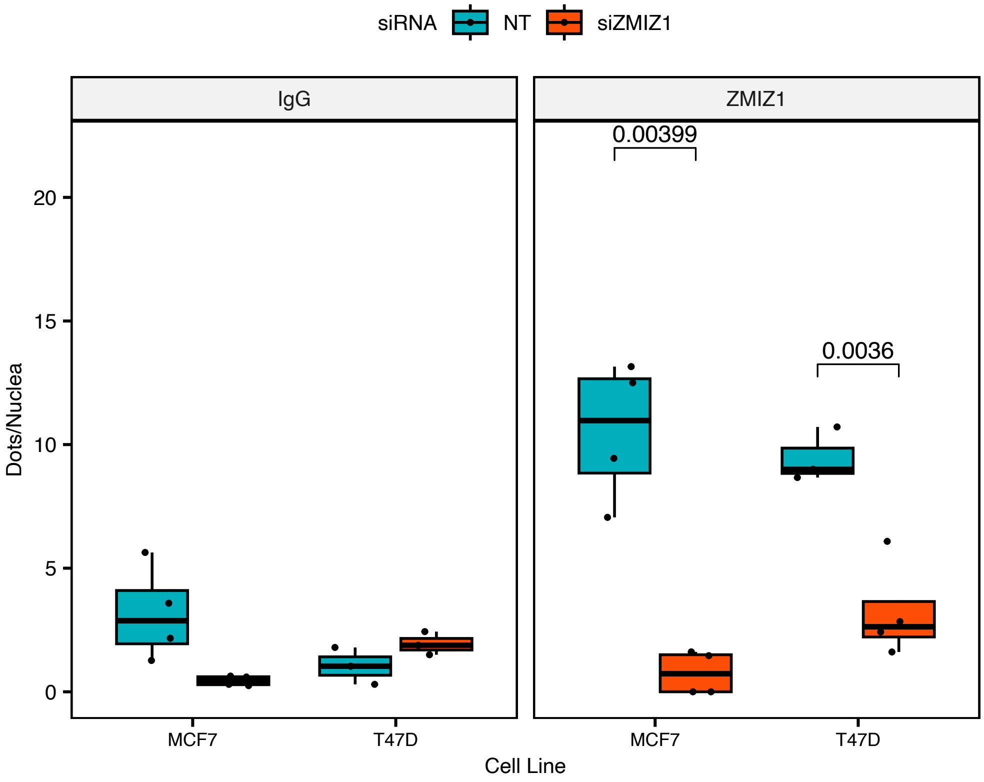

Figure S2: **ZMIZ1 protein control for ER-ZMIZ1 proximity ligation assay (PLA)**.To confirm that the ER-ZMIZ1 PLA signal was dependent on the presence of ZMIZ1 protein, we undertook a further PLA using the SMARTpool on-target siZMIZ1 along with the matched siCTRL. On knockdown of the ZMIZ1 protein, the PLA signal was significantly reduced for both MCF7 and T47D cells. For our ER-IgG control, there was no significant effect. All statistical analysis was conducted using a Student's t-test followed by Bonferroni adjustment for multiple comparisons.

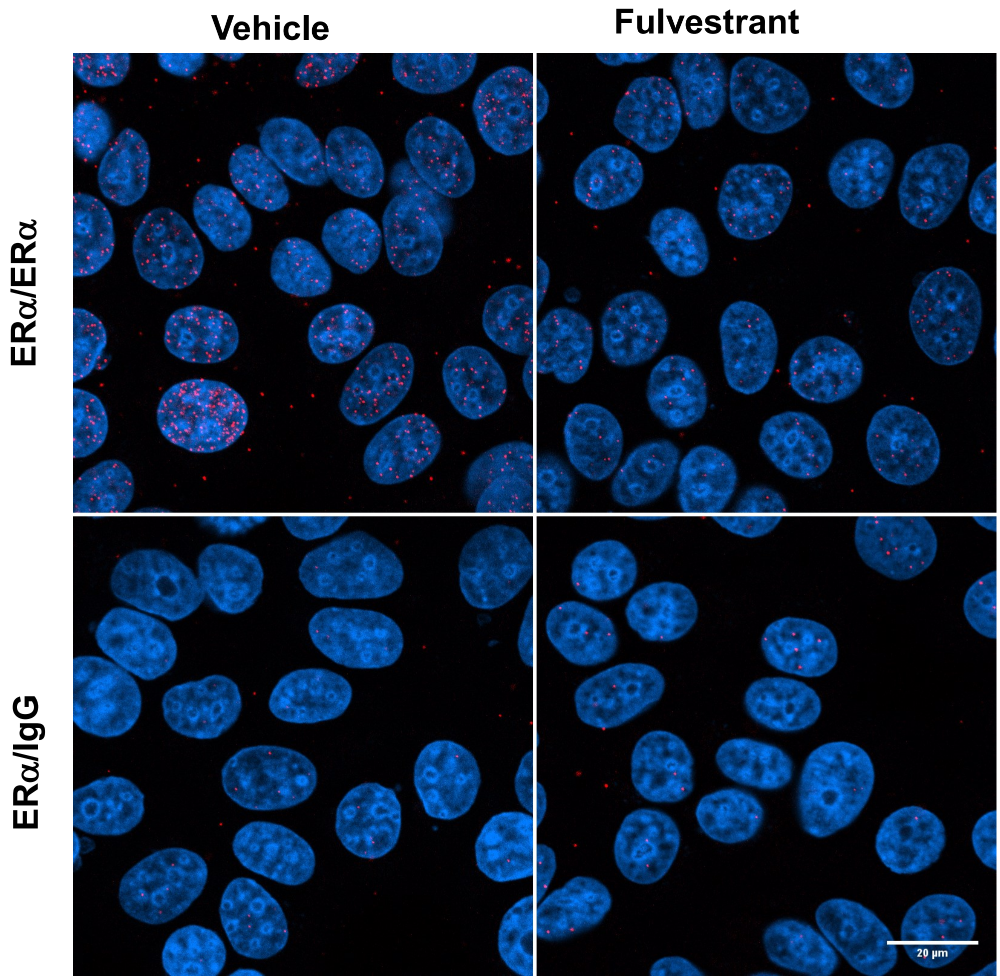

Figure S3: **Biological controls to validate ER-associated interactions in the PLA in MCF7**. MCF7 cells were treated for 24 hours with vehicle (ethanol) or with 100 nM Fulvestrant and were subsequently analysed by PLA. Dual antibody recognition of ER was used as a positive control, and antibodies against ER and an isotype IgG were used together as a negative control. Fulvestrant was added as an additional biological control as it is known to degrade ER. Images were taken at 630x magnification. Cell nuclei are shown in blue and PLA signals are shown as red dots.

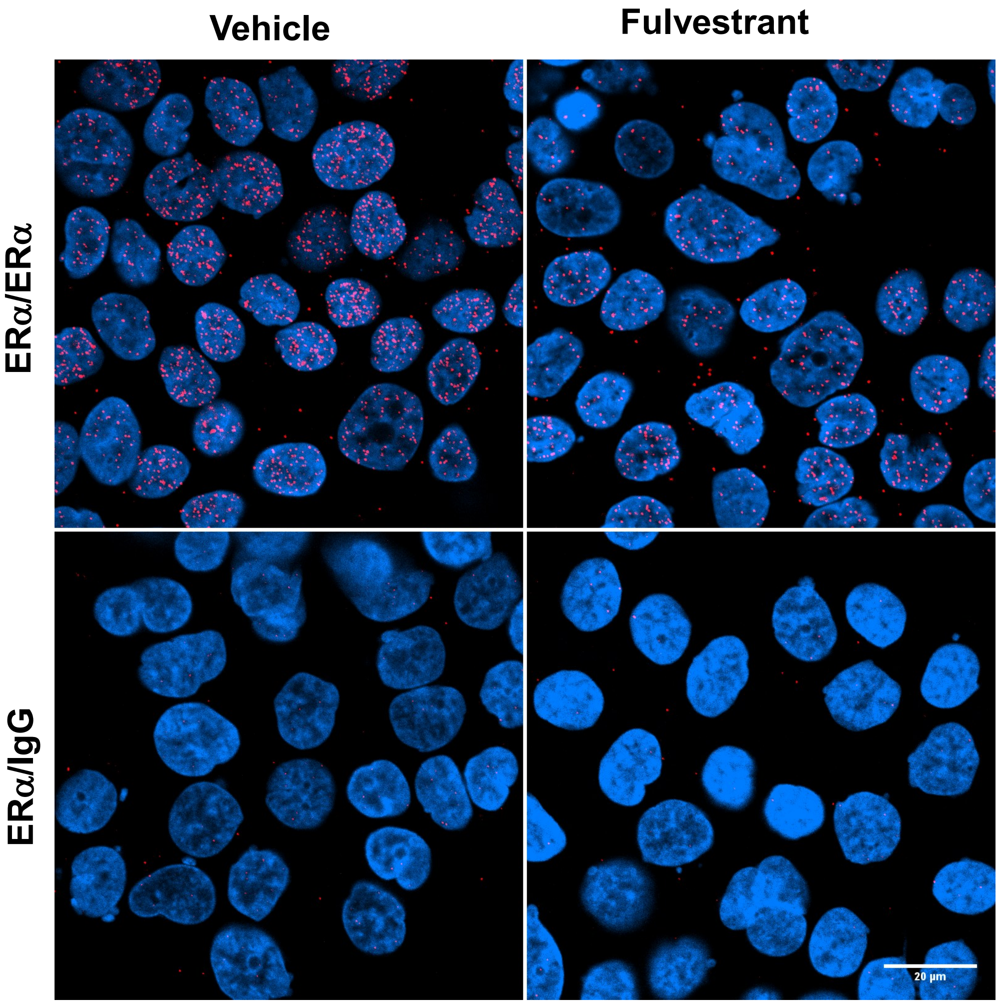

Figure S4: **Biological controls to validate ER-associated interactions in the PLA in T47D**. T47D cells were treated for 24 hours with vehicle (ethanol) or with 100 nM Fulvestrant and were subsequently analysed by PLA. Dual antibody recognition of ER was used as a positive control, and antibodies against ER and an isotype IgG were used together as a negative control. Fulvestrant was added as an additional biological control as it is known to degrade ER. Images were taken at 630x magnification. Cell nuclei are shown in blue and PLA signals are shown as red dots.

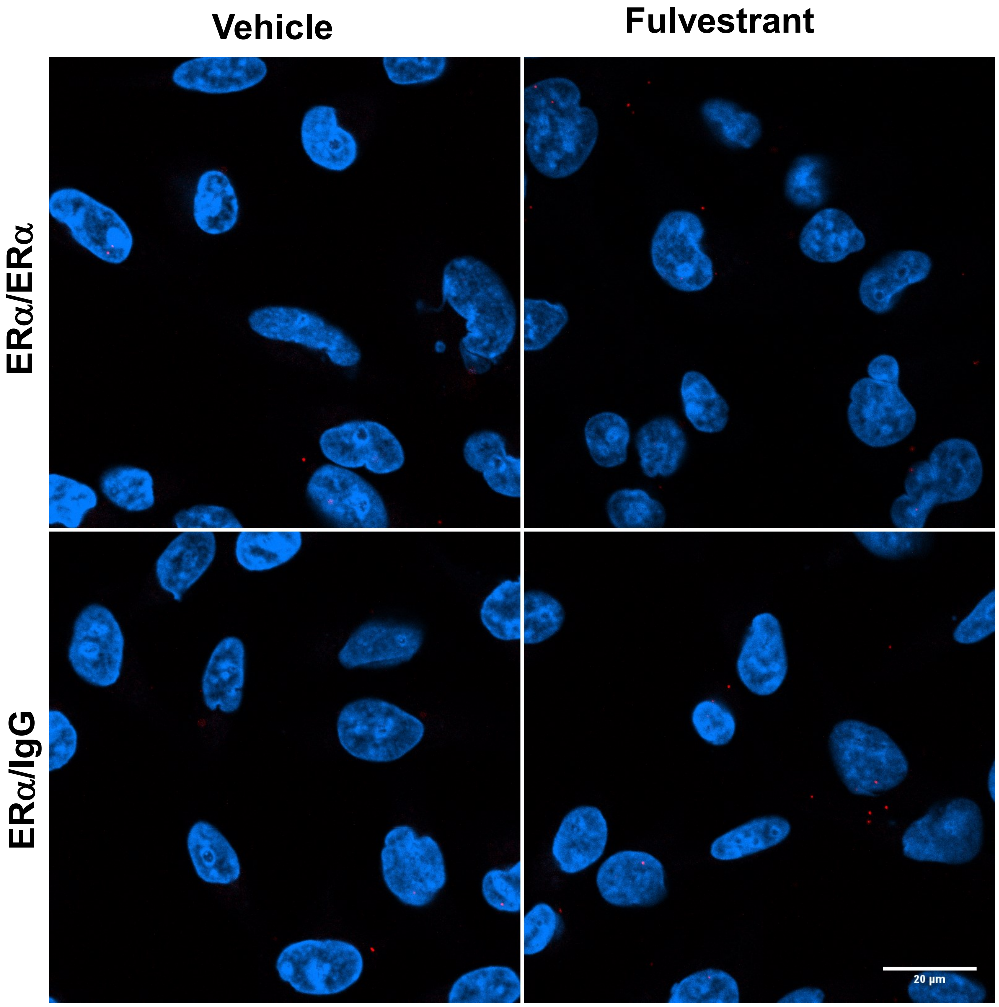

Figure S5: **Biological controls to validate ER-associated interactions in the PLA in MDA-MB-231**. MDA-MB-231 cells were treated for 24 hours with vehicle (ethanol) or with 100 nM Fulvestrant and were subsequently analysed by PLA. Dual antibody recognition of ER was used as a positive control and antibodies against ER and an isotype IgG were used together as a negative control. Fulvestrant was added as an additional biological control as it is known to degrade ER. Images were taken at 630x magnification. Cell nuclei are shown in blue and PLA signals are shown as red dots.

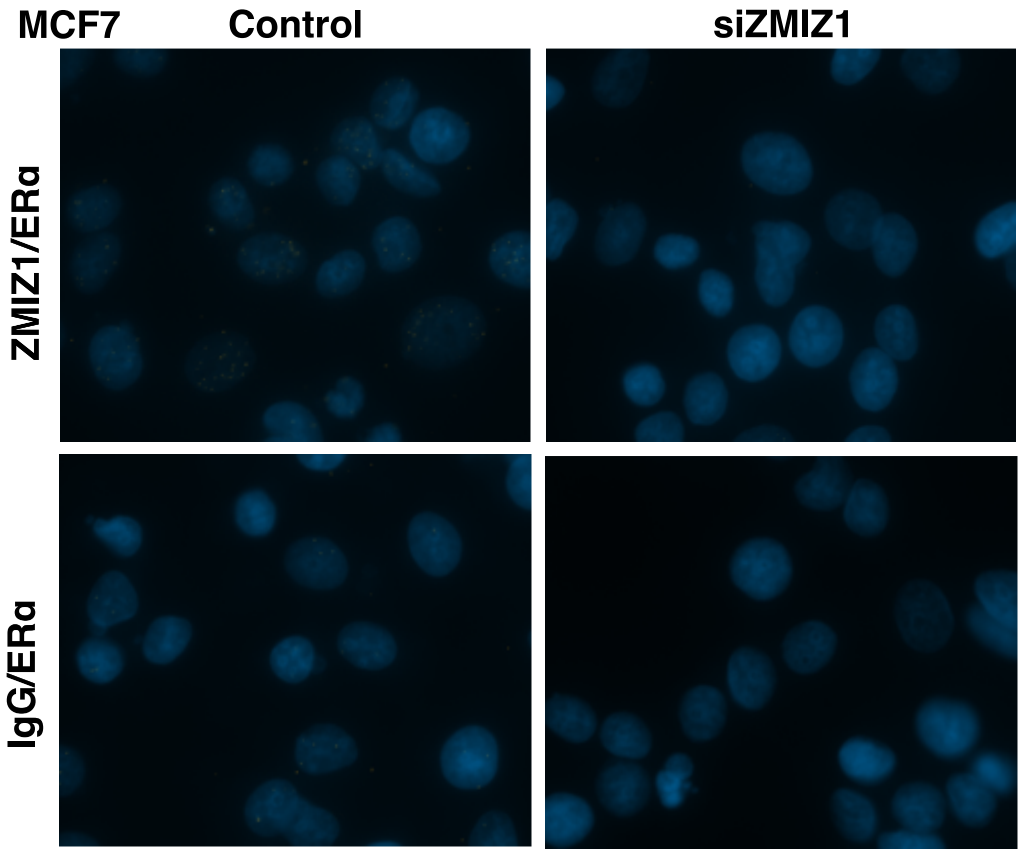

Figure S6: **Representative images of +/-siZMIZ1 PLA in MCF7 cells.** Cells were transfected with either siZMIZ1 targeting siRNA SMARTpool or matched siRNA control. IgG/ERα was included as a negative control. Minimal PLA signal was found for all samples with the exception of the ZMIZ1/ERα control conditions, confirming that the ZMIZ1/ERα signal was dependent on both the ZMIZ1 antibody and the ZMIZ1 protein in MCF7 cells.

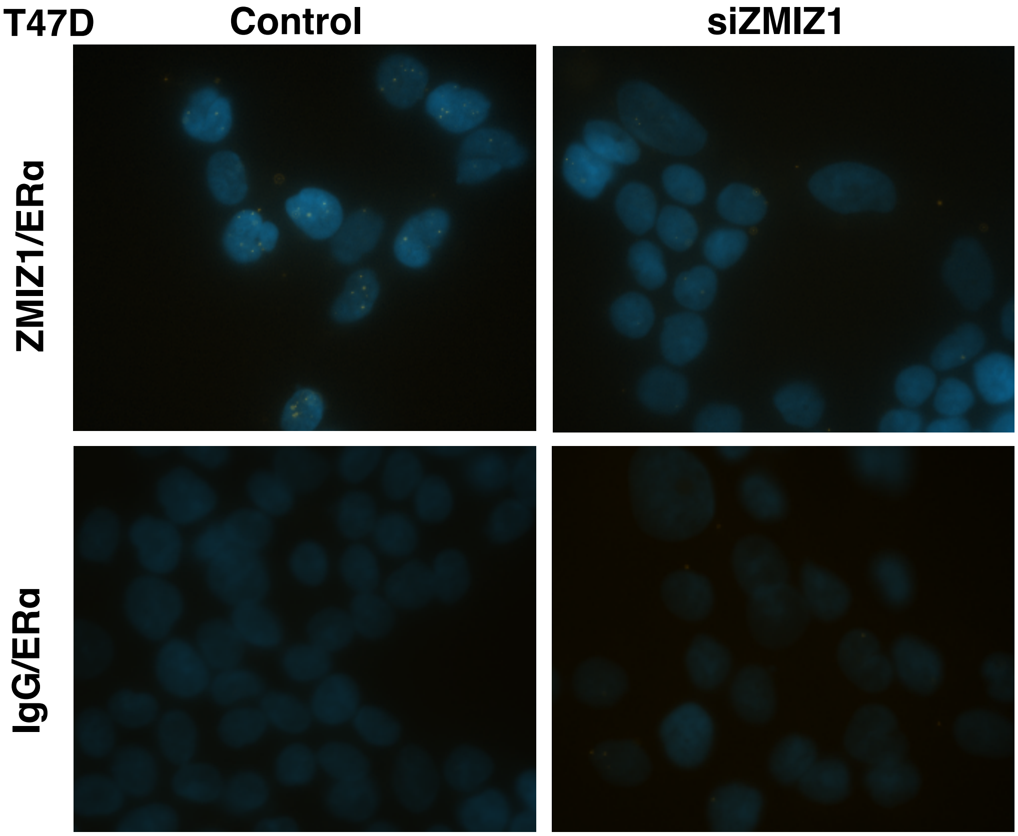

Figure S7: **Representative images of +/-siZMIZ1 PLA in T47D cells.** Cells were transfected with either siZMIZ1 targeting siRNA SMARTpool or matched siRNA control. IgG/ERα was included as a negative control. Minimal PLA signal was found for IgG/ERα samples. The ZMIZ1/ERα siRNA treated cells show significant fewer dots per cell than the siControl samples, confirming that the ZMIZ1/ERα signal was dependent on both the ZMIZ1 antibody and the ZMIZ1 protein in T47D cells.

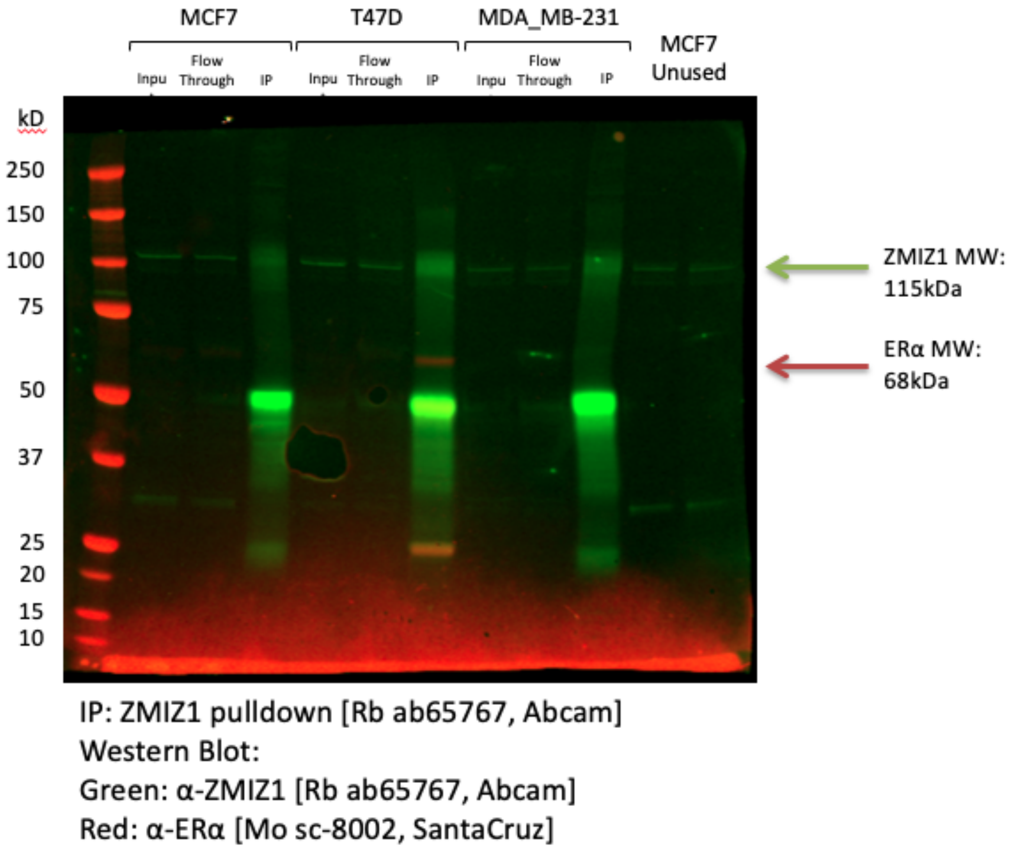

Figure S8: **Co-immunoprecipitation of Estrogen Receptor-α by pull-down of ZMIZ1 from cell lysate (complete blot)**. ZMIZ1 was detected in the input, flow-through (FT) and IP for all three cell lines. The co-IP detected an interaction between ER and ZMIZ1 in the T47D cell line. The lack of detection in MCF7 can be explained by the use of native conditions without cross-linking. Cross-linking in the qPLEX-RIME protocol is used to enhance the detection transient and through DNA interactions. ZMIZ1 was not detected in any ER pull-down co-IPs. ZMIZ1 co-IP and detection was undertaken using ab65767 (Abcam) and ER detection was undertaken with sc-8002 (Santa Cruz). Nonspecific 50 kDa and 17 kDa bands are present on the manufacturer's data sheet for the ZMIZ1 antibody.

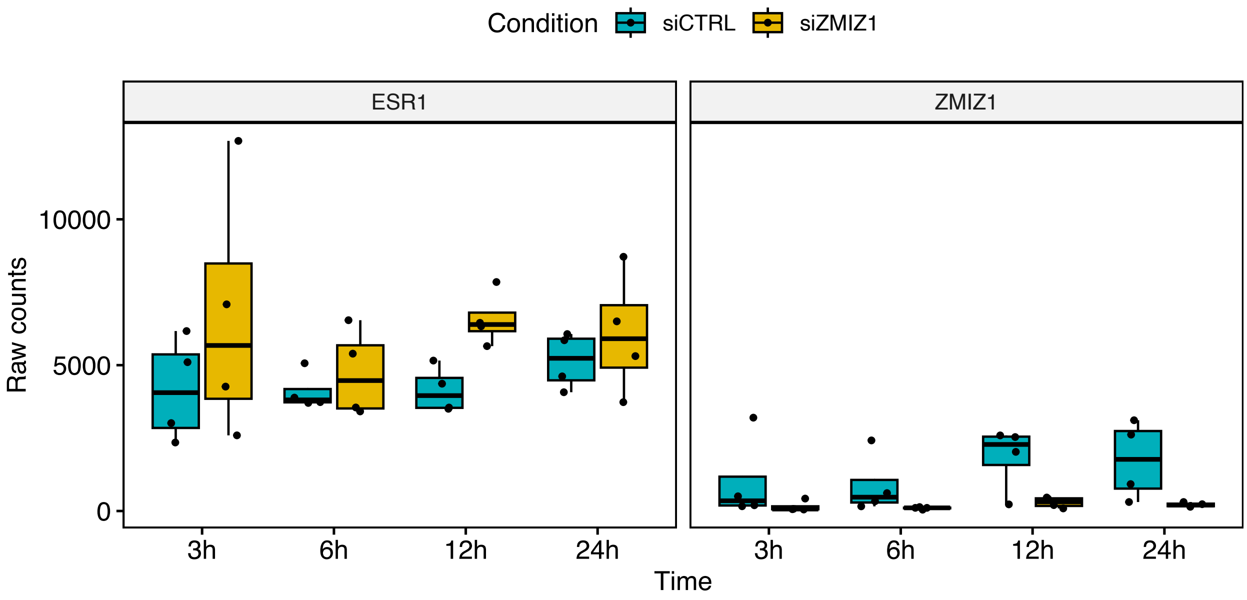

Figure S9: **Knockdown of ZMIZ1 by siRNA in MCF7 cells was detected at all time points**. Plot shows the number of raw reads aligned to ZMIZ1 and ESR1 in sample at each time point. Analysis of ZMIZ1 expression at all time points by DeSEQ2 found the transcript significantly reduced in expression ($p < 0.01$). ER expression was not found to change significantly on ZMIZ1 knockdown at any time point.

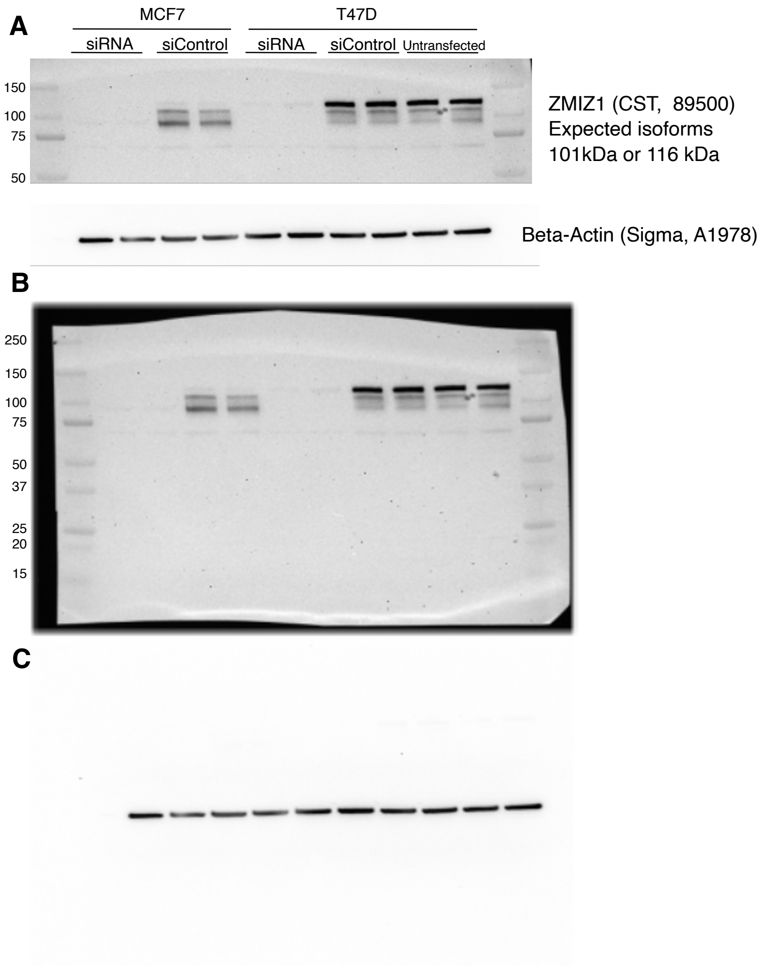

Figure S10: **siRNA knockdown of ZMIZ1 protein was confirmed in MCF7 and T47D.** (A) Treatment with ZMIZ1-targeting siRNAs resulted in near complete loss of the ZMIZ1 related bands in both MCF7 and T47D cell lines, controlled by Beta-Actin. Two isoforms are reported in UniProt, 101 kDa and 116 KDa; however, as ZMIZ1 is also SUMOylated, which has a shift of 15–17 kDa on SDS-Page, (Hilgarth & Sarge, 2005) it is not possible to confirm from the blot if the cell lines express different isoforms or are modified differently. Analysis of RNA-seq data found no evidence of expression of different isoforms (not shown). Full scans of blots shown in (B) and (C).

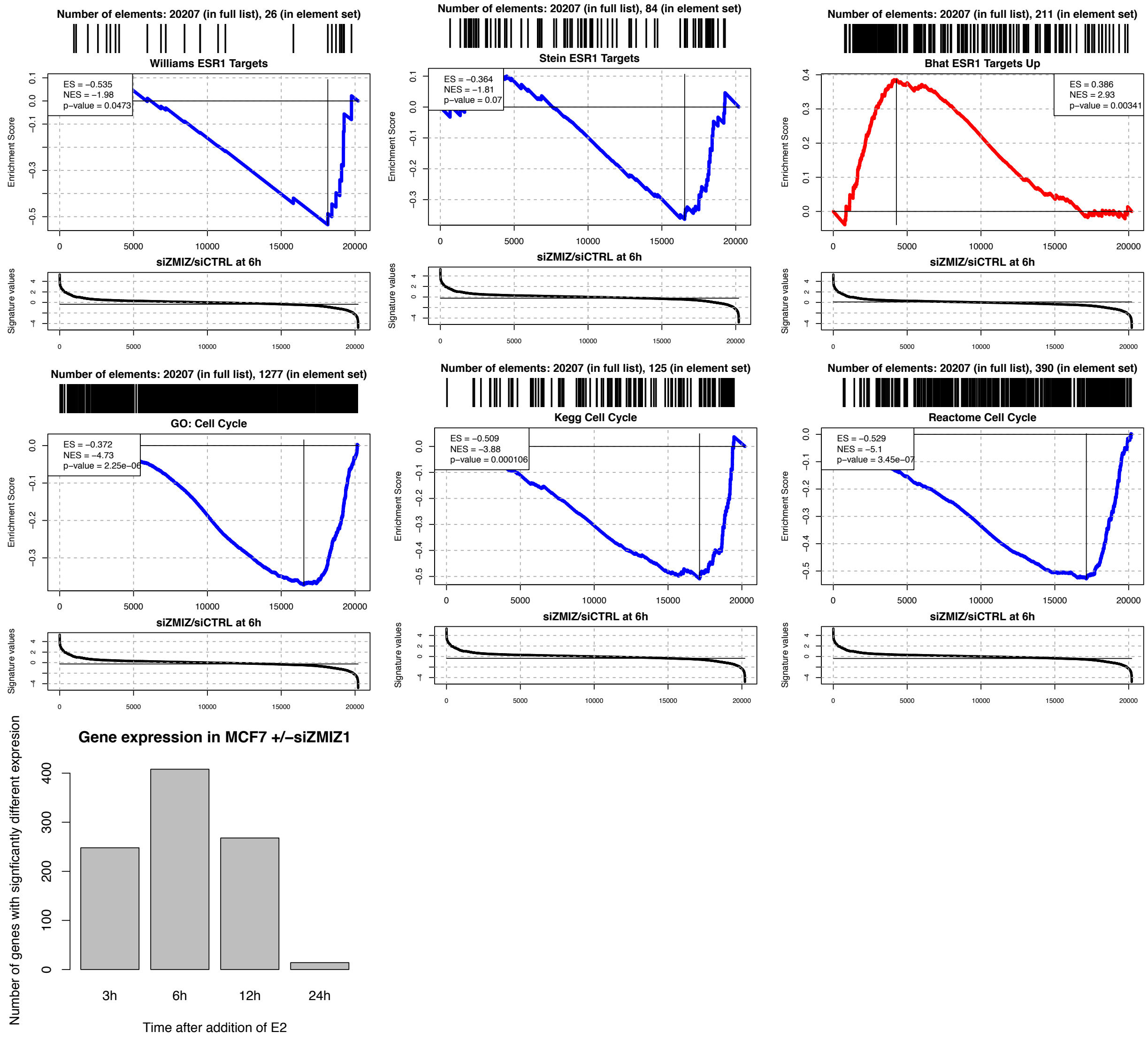

Figure S11: **ZMIZ1 knockdown delays response to E2 in ER regulated cell cycle related genes.** GSEA analysis of RNA-seq +/-siZMIZ1. Row 1–2, GSEA of differentially expressed genes +/-siZMIZ1 at 6 hours after addition of E2. Row 3, Number of differentially expressed genes +/-siZMIZ1 at 3, 6, 12 and 24 hours after addition of E2.

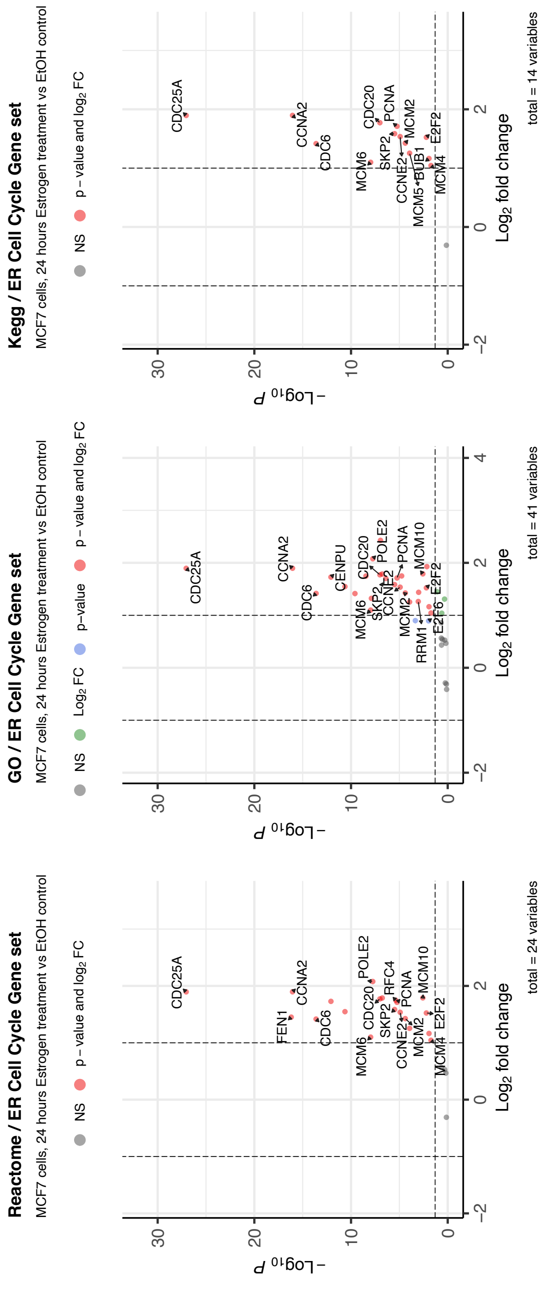

Figure S12: **Reactome, GO and KEGG Cell cycle / ER Response gene sets, are estrogen responsive.** Volcano plots of all three gene sets generated in this study, comparing the expression of genes in Table S2 at 24 hours after treatment with either estrogen or vehicle control (EtOH).

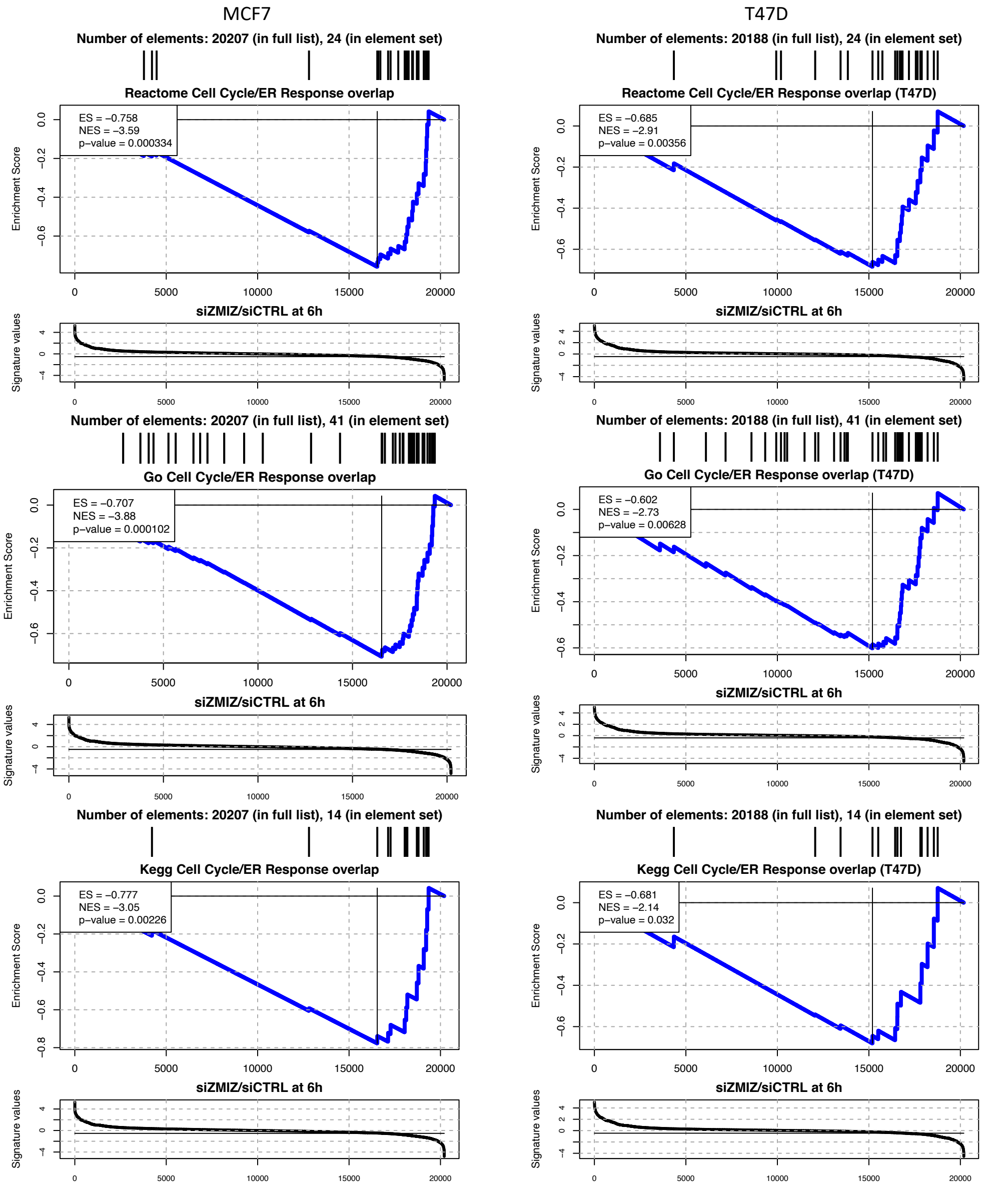

Figure S13: **ZMIZ1 knockdown delays response to E2 in ER regulated cell cycle related genes (Continued).** GSEA of cell cycle specific ER responsive genes shows that knockdown of ZMIZ1 targets these genes in both MCF7 and T47D cell lines. Gene lists for gene sets are provided in Table S2.

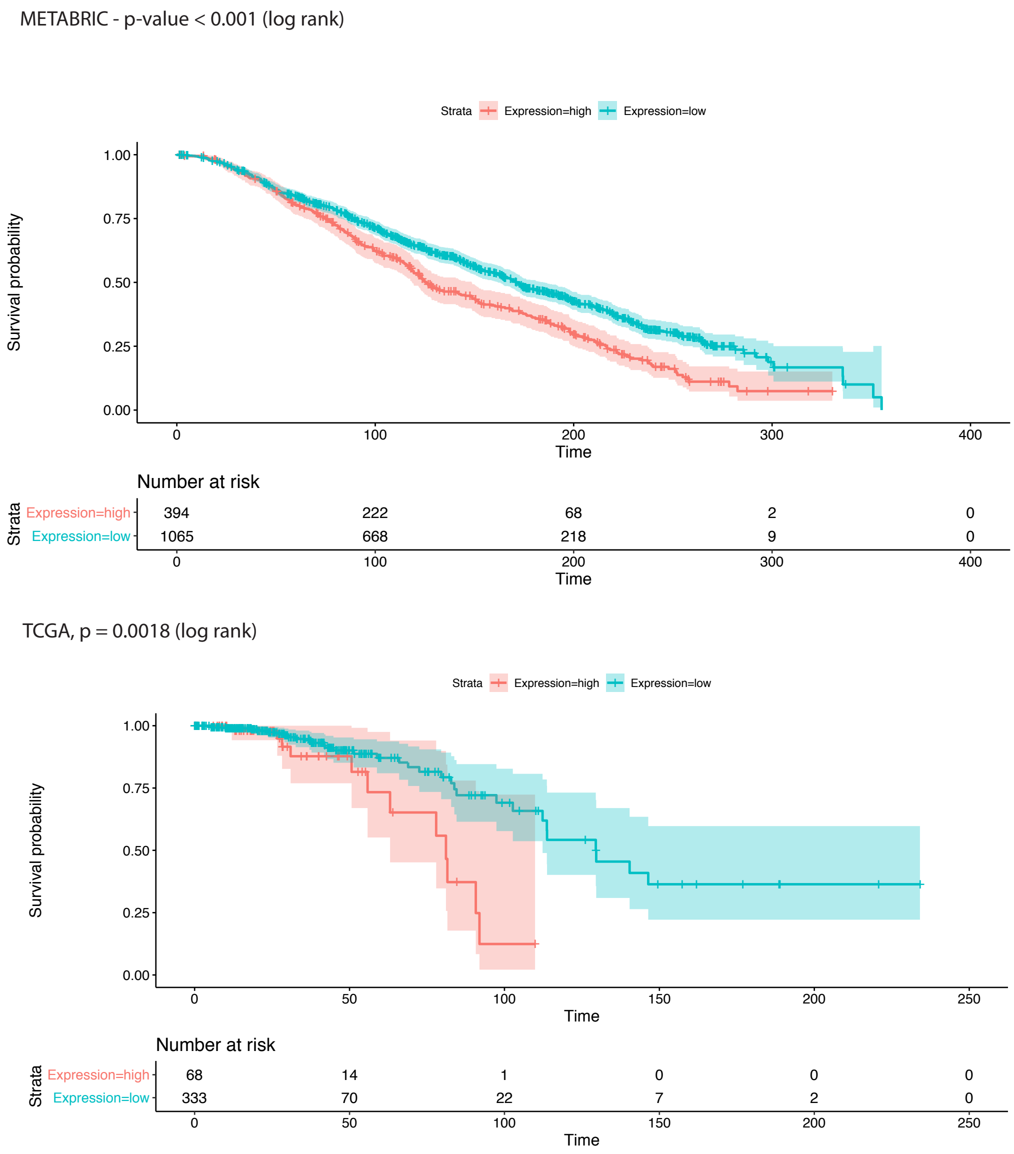

Figure S14: **ZMIZ1 is a significant predictor of survival for ER-positive patients in the METABRIC and TCGA cohorts.** The METABRIC and TCGA cohorts were filtered on ER status as recorded in their respective metadata. The most suitable cut-off for expression was calculated using "survminer" package for R. In both cohorts, ZMIZ1 was a significant predictor of ER-positive patient survival (METABRIC $p < 0.001$, TCGA $p = 0.0018$).

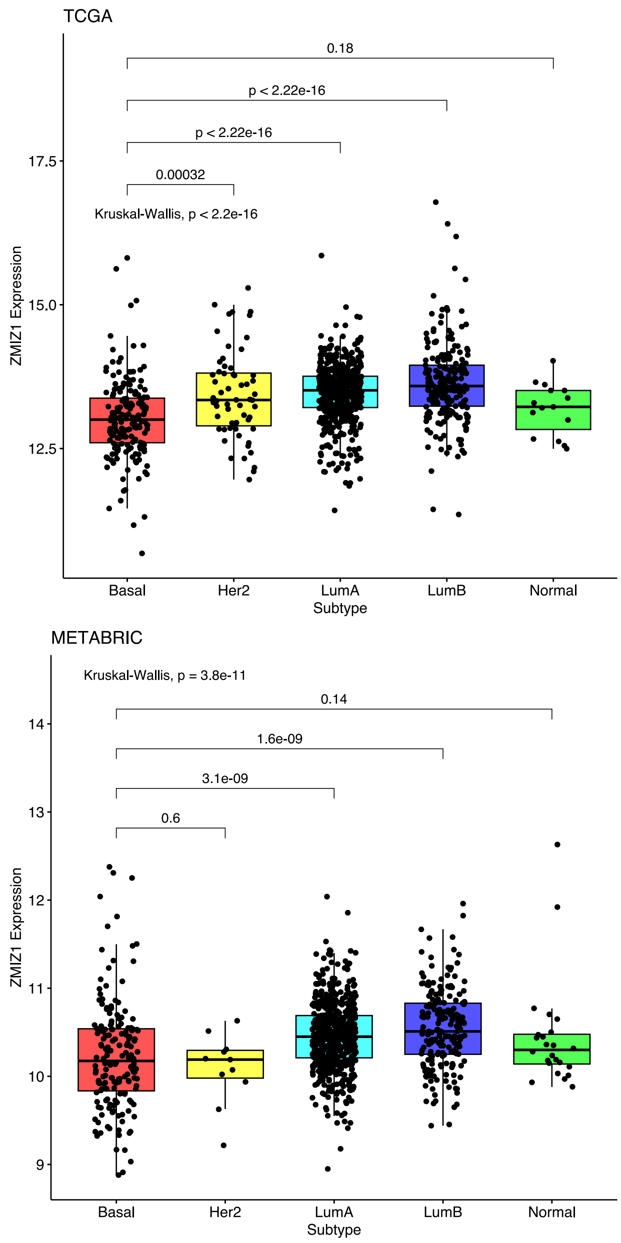

Figure S15: **ZMIZ1 is significantly more expressed in the luminal A and B sub-types than in basal breast cancer.** Expression of ZMIZ1 in both TCGA and METABRIC showed a significant increase in ZMIZ1 expression in the luminal sub-types over basal tumour expression. A significant increase in ZMIZ1 expression was also seen between the Her2 sub-type and Basal sub-type in the TCGA cohort, but this result was not recapitulated in the METABRIC cohort.
